# Genome-Wide Identification and Development of LTR Retrotransposon-Based Molecular Markers for the *Melilotus* Genus

**DOI:** 10.3390/plants10050890

**Published:** 2021-04-28

**Authors:** Zifeng Ouyang, Yimeng Wang, Tiantian Ma, Gisele Kanzana, Fan Wu, Jiyu Zhang

**Affiliations:** The State Key Laboratory of Grassland Agro-Ecosystems, Key Laboratory of Grassland Livestock Industry Innovation, Ministry of Agriculture and Rural Affairs, College of Pastoral Agriculture Science and Technology, Lanzhou University, Lanzhou 730020, China; ouyzf19@lzu.edu.cn (Z.O.); wangym19@lzu.edu.cn (Y.W.); matt17@lzu.edu.cn (T.M.); kanzana16@lzu.edu.cn (G.K.); wuf15@lzu.edu.cn (F.W.)

**Keywords:** *Melilotus*, LTR retrotransposons, genome, polymorphisms, transferability

## Abstract

*Melilotus* is an important genus of legumes with industrial and medicinal value, partly due to the production of coumarin. To explore the genetic diversity and population structure of *Melilotus*, 40 accessions were analyzed using long terminal repeat (LTR) retrotransposon-based markers. A total of 585,894,349 bp of LTR retrotransposon sequences, accounting for 55.28% of the *Melilotus* genome, were identified using bioinformatics tools. A total of 181,040 LTR retrotransposons were identified and classified as *Gypsy*, *Copia*, or another type. A total of 350 pairs of primers were designed for assessing polymorphisms in 15 *Melilotus albus* accessions. Overall, 47 polymorphic primer pairs were screened for their availability and transferability in 18 *Melilotus* species. All the primer pairs were transferable, and 292 alleles were detected at 47 LTR retrotransposon loci. The average polymorphism information content (PIC) value was 0.66, which indicated that these markers were highly informative. Based on unweighted pair group method with arithmetic mean (UPGMA) dendrogram cluster analysis, the 18 *Melilotus* species were classified into three clusters. This study provides important data for future breeding programs and for implementing genetic improvements in the *Melilotus* genus.

## 1. Introduction

Transposons, including retrotransposons or DNA transposons, are mobile genetic elements that are common in the genomes of eukaryotes [1]. A single, open reading frame (ORF) in which Gag and Pol are fused is common to both Ty1-copia and Ty3-gypsy elements, although many retroelements have an extra ORF with an unknown function [2]. Long terminal repeat (LTR) retrotransposons that possess two long terminal repeats are the most abundant group of transposons in plants [3], and they are particularly abundant in species with large genomes [4]. Moreover, LTR retrotransposons play key roles in plant phenotype variations and in the evolution of genome structure and function [5,6,7,8]. Mascagni et al. studied the relationship between changes in LTR retrotransposon abundance and the evolution of a genus and confirmed that LTR retrotransposons have continued to evolve during speciation [9]. Barghini et al. investigated LTR retrotransposon dynamics in the evolution of the olive (*Olea europaea*) genome and found that retrotransposon activity has impacted the olive genome structure in more ancient times than in other angiosperms [10]. *Copia* and *Gypsy* are the two main subgroups of LTR retrotransposons, and the major structural difference between the *Copia* and *Gypsy* groups is based on the order of reverse transcriptase (RT) and integrase [11].

Given the abundant, ubiquitous, and transcriptionally active retrotransposons in plant genomes, many molecular marker systems have been developed to exploit insertional polymorphisms [12]. Several molecular barcoding methods have been developed for LTR retrotransposons on the basis of PCR technology, including the use of retrotransposon-microsatellite amplified polymorphism (REMAP), retrotransposon-based insertion polymorphism (RBIP), inter-primer binding sequence (IPBS), inter-retrotransposon amplified polymorphism (IRAP), and sequence-specific amplified polymorphism (SSAP) markers [13]. These developed markers have been deployed in a range of crop and wild plant species. Previous studies of the genetic diversity and evolution of field pea (*Pisum*), as revealed by RBIP analysis, grouped 3020 *Pisum* germplasms into landraces, cultivars, and wild *Pisum* [14].

*Melilotus* presents industrial value for coumarin production and is a highly important legume crop that includes annual or biennial types. Moreover, it is an extremely important green manure crop for agriculture and animal husbandry and is distributed mainly in the northern part of China [15]. Compared to most other forage types, *Melilotus* tends to show tolerance to harsh environmental conditions and exhibits high seed yields [16,17]. As a forage legume, *Melilotu*s possesses the ability to fix nitrogen in a symbiotic interaction with soil rhizobia [18] and shows excellent nitrogen production, making these plants useful for crop rotations [19]. Because of the biological activity of its flavones, coumarins, and saponin, *Melilotus* (also known as wild alfalfa) is used in traditional Tibetan medicine and is grown as a honey plant [15,20], but high coumarin content can prohibit the use of this genus for forage. *Melilotus* has an extremely high market value and is widely used in Chinese herbal medicine, so studying the genetic diversity of *Melilotus* genus is a worthy endeavor [21]. Additionally, genetic studies of *Melilotus* are required to better understand the extent of interspecific variations in the genus. In general, molecular markers are extremely useful in assessing genetic diversity and identifying novel genotypes among the *Melilotus* germplasm. However, LTR retrotransposon markers, which are excellent and sensitive tools for detecting genetic diversity and rapid genome changes, still have not been used to study *Melilotus*. Thus, the identification and study of LTR elements are two of the basic and indispensable steps for understanding the biology and evolution of this genus [22]. Our research group completed the whole-genome sequencing of diploid *M. albus*, with a genome size of approximately 1.04 Gb (BioProject ID: PRJNA674670) and eight chromosomes (2n = 16) [23]. In all, 772,285 transposable elements (TEs) and 181,040 LTR-RTs (retrotransposons) were identified. The LTR-RTs accounted for 55.28% of the *Melilotus* genome, which is higher than the value determined from the *Medicago* *truncatula* genomes [24]. Neutrality is a desirable feature in evaluations of genetic differences between populations, so we identified outlier loci and performed neutrality tests on the developed markers. Herein, we aimed to identify and develop LTR retrotransposon markers in the *Melilotus* genome on the basis of the above data to assess the population structure and genetic diversity in other members of the genus.

## 2. Results

### 2.1. Identification and Analysis of LTR Retrotransposons in the Melilotus Genome

The number of identified LTR-RTs was 181,040 in the *Melilotus* genome (Table S1), with 101,240 belonging to the *Ty3-gypsy* group, 77,935 belonging to the *Ty1-copia* subgroup, and 1865 belonging to the other subgroup. In all, 168,428 LTR-RTs were successfully mapped to eight chromosomes of *Melilotus*. The maximum LTR-RTs was found on chromosome 2 (∼1.38%), and the minimum was observed on chromosome 4 (∼1.18%) (Figure S1). Their total length was 585,894,349 bp, which accounted for 55.28% of the *Melilotus* genome. In total, 350 primer pairs were designed on the basis of RBIPs, IRAPs, ISBPs, and REMAPs (Table 1).

### 2.2. Amplification with LTR Primers in Melilotus

**Figure 1:**
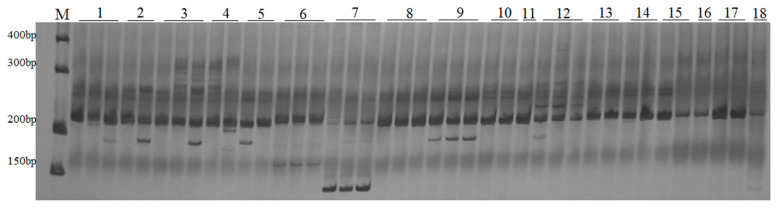
DNA amplification with primer 95 in genotyping of 18 *Melilotus* species process.

**Figure 2:**
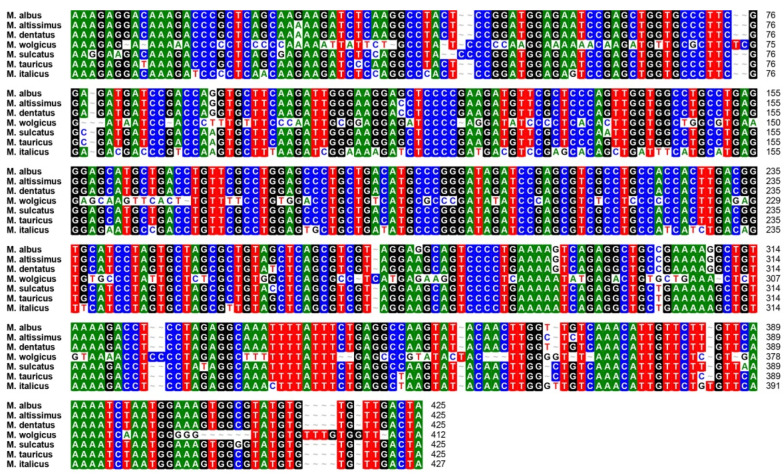
Multiple sequence alignment of PCR products of LTR_RT_302 primer in seven *Melilotus* species.

To amplify the genomic DNA of four accessions (Acc3, Acc5, Acc6, and Acc7) that were randomly selected for primer identification, 350 LTR-RT primer pairs were initially used. The total number of primer pairs that generated amplification products was 320, and 79 primer pairs showed polymorphism in four accessions. The polymorphic primers were chosen for further screening using 15 *M. albus* accessions, and each accession included four individual plants (Figure S2). Among the primer pairs, 47 produced bands and revealed polymorphisms, including 34 pairs of RBIP primers and 13 pairs of IRAP primers (Figure 1, Table S2). Additionally, to confirm the authenticity and accuracy of the PCR amplification bands obtained, the electrophoretic bands produced by primer pair Ma_LTR_302, which amplified variant alleles ranging from 411 to 426 bp in the seven *Melilotus* species, were sequenced. The primer pair Ma_LTR_302 belongs to the RBIP type, with the forward primer located outside of the LTR in the surrounding genome sequence of the LTR-RTs and the reverse primer located in the LTR regions of the LTR-RTs (Figure 2). We found that the flanking sequence inserted by LTR-RT is the main cause of polymorphisms, which is in accordance with previous studies [12].

### 2.3. Transferability of the Newly Developed LTR Retrotransposon-Based Markers

In the first step, 79 pairs of primers were selected and further screened in 15 *M. albus* accessions. In total, 47of the 79 LTR primer pairs showed polymorphisms among 15 *M. albus* accessions. A total of 182 alleles were obtained, and 3 to 7 alleles were observed per locus, with an average of 3.96. Primer 229 had the largest number of polymorphic bands (7) and highest polymorphism information content (PIC) value. The average expected heterozygosity (He) was 0.61, ranging from 0.42 to 0.8. The PIC values were between 0.38 and 0.77, with a mean of 0.54 (Table 2).

In this study, 47 newly developed LTR-RT markers generated polymorphic bands in 40 accessions of 18 *Melilotus* species. A total of 292 alleles were obtained at the 47 transferable LTR-RT-based markers in 18 species, and 3 to 15 alleles were observed per locus, with an average of 6.21 (Table S2), and 265 polymorphic loci were observed (90.75%). Primer 62 had the largest number of polymorphic bands (15) and highest PIC value, while primers 152, 277, and 290 produced the lowest number of polymorphic bands (3). The average He was 0.70, ranging from 0.37 (primer 93) to 0.86 (primer 283). The PIC values were between 0.35 (primer 93) and 0.84 (primer 283), with a mean of 0.66.

### 2.4. Outlier Detection

**Figure 3:**
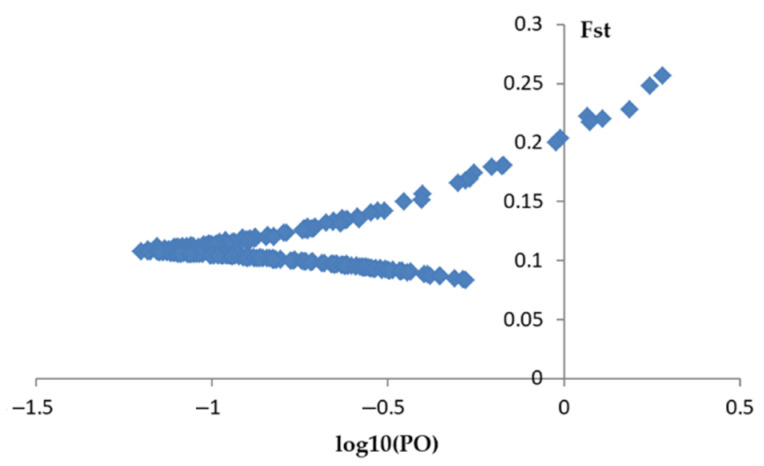
BayeScan plot of 47 polymorphic LTR-RT markers in 18 *Melilotus*. F_st_ is plotted against the log10 of the posterior odds (PO).

We successfully tested a total of 47 polymorphic LTR-RT markers in 18 *Melilotus* populations. We used the BayeScan 2.1 program and revealed 14 outlier loci in the group of 292 amplified loci (Figure 3). All the detected outlier loci had positive alpha values and high *F_ST_* values (*F_ST_* = 0.16771–0.25660; Table S3).

### 2.5. Genetic Diversity

The percentage of polymorphic loci (PPL), number of polymorphic loci (NPL), effective number of alleles (Ne), observed number of alleles (N_A_), Shannon’s information index (I), and Nei’s (1973) gene diversity (h) varied among the *Melilotus* species (Table 3). The NPL ranged from 0 (*M. sulcatus*, *M. segetalis,* and *M. wolgicus*) to 98 (*M. italicus*), with a mean of 37.67, and the highest and lowest PPL were 33.56% and 0%, respectively, with a mean of 12.90. The N_A_ ranged between 1 (*M. segetalis*, *M. sulcatus,* and *M. wolgicus*) and 1.3356 in *M. italicus*, with an average value of 1.1290. The Ne varied from 1 (*M. segetalis*, *M. sulcatus,* and *M. wolgicus*) to 1.2282 (*M. italicus*), with an average of 1.0879. The highest level of h (0.1309) was observed for *M. italicus*, while the lowest level of h (0) was recorded for *M. segetalis*, *M. sulcatus,* and *M. wolgicus*, with an average value of 0.0510. Accordingly, the average I was 0.0750.

### 2.6. Cluster and Population Structure Analysis

**Figure 4:**
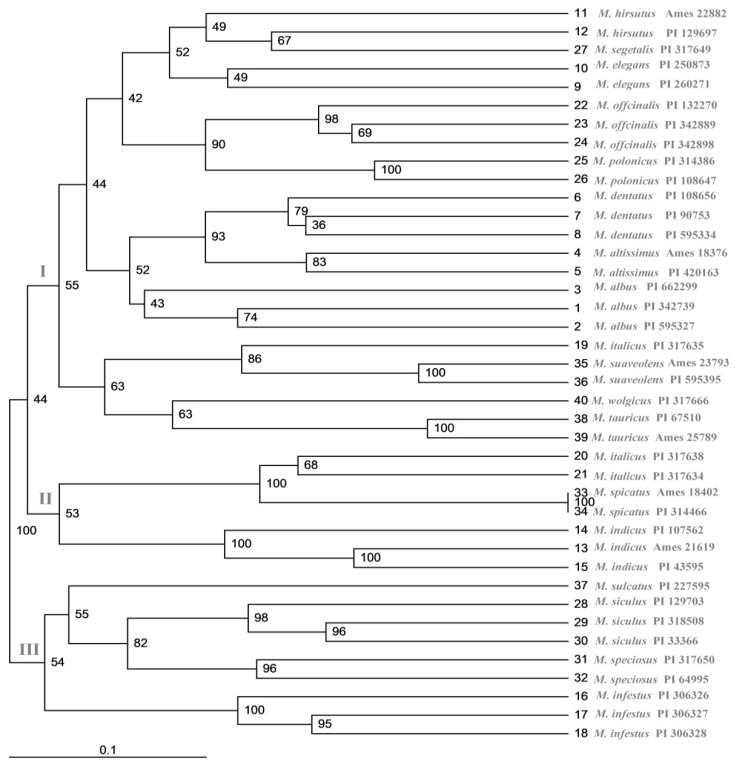
Dendrogram of 18 *Melilotus* by the UPGMA cluster analysis based on the LTR-RT analysis. The numbers present inside the clusters represent the bootstrap values. The numbers in bold represent different *Melilotus* species as defined in Table 5.

The unweighted pair group method with arithmetic mean (UPGMA) dendrogram showed that the 18 *Melilotus* species were divided into three clusters (Figure 4). Cluster I included *M. elegans*, *M. dentatus*, *M. albus*, *M. hirsutus*, *M. altissimus*, *M. segetalis*, *M. officinalis*, *M. polonicus*, *M. suaveolens*, *M. tauricus,* and *M. wolgicus*, except for germplasm PI317635 of *M. italicus*. Cluster II contained four species, namely, *M. infestus*, *M. speciosus*, *M. sulcatus,* and *M. siculus*. Cluster III contained the remaining three species, namely, *M. italicus*, *M. indicus,* and *M. spicatus*. In the dendrogram constructed, bootstrap values ranged from 36% to 100% between clusters, and the average bootstrap value observed in this study was 75%.

Cophenetic correlation analysis was carried out to confirm the grouping pattern of the *Melilotus* species (Figure S3). The correlation test results showed that the correlation coefficient r was equal to 0.817 (Figure S3B).

Furthermore, 15 individual plants of *M. albus* were classified into four main clusters (Figure S4). The cluster analysis showed that single plants from an accession were clustered together, and the genetic similarity coefficients of 15 germplasms ranged between 0.75 and 0.95, thus revealing their close genetic relationships.

**Figure 5:**
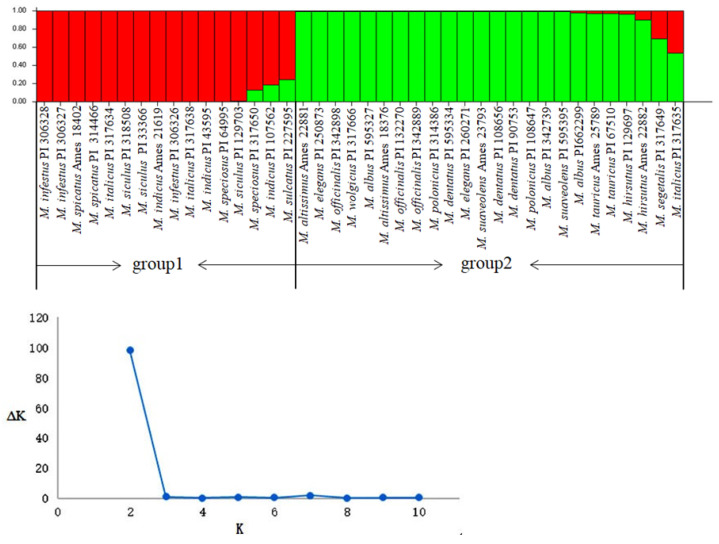
Genetic structure of 40 accessions for 18 *Melilotus* species as inferred by STRUCTURE with 47 LTR-RT markers.

In accordance with the observed optimal goodness of fit (K = 2), the Bayesian clustering model, which was carried out with STRUCTURE software on all individuals and run for K = 1–11, divided the 40 evaluated accessions belonging to 18 *Melilotus* species into two groups (Figure 5). Group 1 contained 16 individuals belonging to 7 species, namely, *M. sulcatus*, *M. siculus*, *M. spicatus*, *M. speciosus*, *M. italicus*, *M. infestus*, and *M. indicus*. Group 2 contained 11 species, namely, *M. altissimus*, *M. albus*, *M. hirsutus*, *M. dentatus*, *M. elegans*, *M. segetalis*, *M. officinalis*, *M. polonicus*, *M. suaveolens*, *M. wolgicus,* and *M. tauricus*, except for germplasm PI317635 of *M. italicus*.

Genetic variation within and among the *Melilotus* were determined by analysis of molecular variance (AMOVA). All species were analyzed to be a single group by AMOVA (Table 4). According to the AMOVA results, there were highly significant differences (*p* < 0.001) in genetic differentiation among species and within species. Of the total genetic variance, 45.19% was due to differences among species, and 54.81% was due to differences within species. Therefore, the results showed significant genetic differences among the 18 *Melilotus* species of the one group.

According to the structure results, we used the analysis of molecular variance (AMOVA) to evaluate variance components among groups, among species within groups and within species (Table 4). According to the AMOVA results, there were highly significant differences (*p* < 0.001) in genetic differentiation among groups, among species within groups and within species. Of the total genetic variance, 12.13% was due to differences among groups, 44.29% was due to differences among species within groups, and the remaining 43.58% was due to differences within species. Therefore, the results showed significant genetic differences among the 18 *Melilotus* species of the two groups.

**Table 4:** Analysis of molecular variance (AMOVA) for 18 *Melilotus* species of the two groups.

Source of Variation	Degrees of Freedom	Sum of Squares	Variance Components	Percentage of Variation	*p*-Value
Among groups	1	167.759	5.198	12.13	<0.001
Among species within groups	16	963.857	18.976	44.29	<0.001
Within species	22	410.833	18.674	43.58	<0.001

**Table 5:** The four classifications of molecular phylogeny, SSR, EST-SSR, and LTR-RT makers in *Melilotus*.

Categories	Classification	Subclassification	Species
Molecular phylogeny [25]	Clade I		*M. albus, M. altissimus, M. hirsatus, M.officinalis, M. polonicus, M. suaveolens, M. wolgicus, M. elegans, M. dentatus, M. tauricus*
	Clade II	Clade 1	*M. spicatus*
		IIb	*M. indicus, M. segetalis*
		Clade 2	*M. infestus*
		IIa	*M. siculus, M. sulcatus, M. speciosus, M. italicus*
SSR makers [26]	A		*M. albus, M. altissimus, M. hirsutus, M. officinalis, M. polonicus, M. suaveolens, M. wolgicus, M. elegans, M.infestus, M. spicatus, M. sulcatus,*
		A1	*M. italicus, M. speciosus*
	B		*M. dentatus, M. siculus, M. tauricus*
		B1	*M. indicus, M. segetalis*
EST-SSR markers [27]	I		*M. albus, M. altissimus, M. hirsutus, M. officinalis, M. polonicus, M. suaveolens, M. wolgicus, M. elegans, M. dentatus, M. tauricus*
	II		*M. indicus, M. segetalis, M. italicus, M. spicatus*
	III		*M. infestus, M. siculus, M. speciosus, M. sulcatus*
LTR-RT markers	GI		*M. albus, M. altissimus, M. hirsutus, M. officinalis, M. polonicus, M. suaveolens, M. wolgicus, M. elegans, M. dentatus, M. tauricus, M. segetalis*
	GIII GII		*M. indicus, M. italicus, M. spicatus* *M. infestus, M. siculus, M. speciosus, M. sulcatus*

## 3. Discussion

Molecular markers based on LTR retrotransposons show broad applications in genetic mapping, genetic diversity assessment [28,29], phylogenetic evolution analysis [30], and variety identification [31]. Additionally, compared with traditional phenotypic markers, molecular markers are more efficient, accurate, and reliable for differentiating varieties and closely related species [32]. Nevertheless, relatively few molecular markers have been found in numerous non-model plants, including *Melilotus*, which considerably limits the genetic research on these species. In previous studies, the genetic diversity of *Melilotus* has been investigated using different molecular markers, such as simple sequence repeats (SSRs) [26,33] and expressed sequence tags-simple sequence repeats (EST-SSRs) [27,34]. In plant research, retrotransposons play a major role in genome evolution [35], and the presence of a very large number of error-prone retrovirus replications can lead to accumulation of genetic variations [36]. Ramakrishnan et al. deployed retrotransposon-based markers to reveal the genetic diversity and population structure of Asian bamboo [37]. However, LTR-RT-based molecular markers, which are remarkable tools in detecting genetic diversity, have not previously been used in *Melilotus.*

In this study, 181,040 LTR retrotransposons were used to develop 350 LTR primer pairs for PCR amplification. In total, 47 of the 79 LTR primer pairs showed polymorphisms among 15 *M. albus* accessions, which indicated that this type of molecular marker can distinguish 15 *M. albus* accessions well (Figures S2 and Figure S3). The high levels of polymorphism observed may be due to the *M. albus* materials selected for screening the primers. In addition, the polymorphic primer pairs were screened to validate the availability and transferability of LTR-RTs in a panel of 40 *Melilotus* accessions, which yielded 292 clear strong bands. The average number of alleles per primer pair found in this study was higher than the number of EST-SSRs [27,38], while 265 (90.75%) of 292 bands were polymorphic and showed the potential for use in genotyping. Moreover, all of the 47 primer pairs could amplify products successfully in most species and showed stable transferability, exhibiting a higher transferability rate than that obtained in chokecherry (*Prunus virginiana* L.) [39] and that from *Melilotus* EST-SSR primers [27]. Molecular markers are feasible for evaluating genetic diversity in plant species [40]. The genetic diversity revealed at LTR-RT loci was supported by high values of He and PIC. In this study, the mean He and PIC were 0.70 and 0.66, respectively. Primer 283 showed a stronger ability to discriminate genotypes due to its high PIC value (0.84). The primer 93 marker showed a lower PIC value (0.35), suggesting that this primer had less discriminatory ability in the present study. These results clearly suggest that more diverse LTR-retrotransposon marker loci can be identified and effectively applied to breeding programs to obtain the plant types desired for commercial cultivation. The I value can be used to evaluate the level of genetic diversity in a population, where the greater the I is, the greater the genetic diversity [41]. Yan et al. [27] obtained a lower average I value (0.0670) than this study (0.0750) using EST-SSR markers, indicating that the *Melilotus* accessions evaluated in this study were more diverse. In addition, the EST-SSR marker analysis did not consider mononucleotide repeats because of the difficulty of distinguishing single nucleotide repeats from polyadenylation products and single nucleotide stretch errors produced by sequencing [27].

In previous molecular phylogenetic analyses [25], 18 *Melilotus* species were classified into clade I and clade II (Table 5). An SSR maker analysis showed that all *Melilotus* species were clustered into A and B [26], which were further divided into A1 and B1, respectively (Table 5). An EST-SSR marker analysis showed that the 18 *Melilotus* species were grouped into I, II, and III [27]. According to the UPGMA cluster analysis involving the LTR-RT markers conducted in this study, the 18 species were grouped into three clusters. Among the 11 species in cluster I, 10, 10 and 8 of the species were consistent with clade I, I and A, respectively. Except for *M. segetalis*, the GIII of the phylogenetic trees was consistent with II. Cluster II contained the remaining species, which was consistent with III. As the primers increased, the results became more similar. *Melilotus* germplasms from different countries clustered together, indicating that kinship has a greater impact on the genetic structure than the location provenance used in the present study.

The detection of natural selection signatures within a genome can reveal which genes are under the influence of natural selection. It is possible to identify loci with an atypical variation pattern (outlier loci) by comparing the genetic diversity of loci across the genome, which is likely to be affected by selection. Outlier loci can better explain the adaptive genetic variation that is not accounted for by neutral loci [42]. Although a large number of loci were revealed in this study, less than 5% were identified as outliers (Table S3). Additionally, the results obtained with the LTR-RT markers were similar to those with the microsatellite (SSR) method, which revealed different sizes of DNA fragments by electrophoresis. Although LTR retrotransposons are highly heterogeneous in plants, homoplasy is also obviously important in evaluating phylogenetic relationships among species. However, microsatellites follow a stepwise mutation process, considering allele size difference, and it may be more suitable to use microsatellite data analysis [43].

## 4. Materials and Methods

### 4.1. Plant Materials, Genomic DNA Isolation, and PCR Primer Design

A total of 15 accessions of *Melilotus albus* were utilized to screen polymorphic LTR-RT-based markers and evaluate genetic diversity (Table S4). In total, 40 accessions of 18 *Melilotus* species were obtained to assess the newly developed LTR-RT-based markers for their transferability (Table 6). The seeds of the accessions utilized in this study were obtained from the National Plant Germplasm System (NPGS, Beltsville, MD, USA). Total genomic DNA was extracted using the sodium dodecyl sulfate (SDS) method [44]. The quality of the isolated DNA was assessed in 1% agarose gels and by a NanoDrop spectrophotometer (ND-1000, Thermo Scientific™, Waltham, MA, USA). The DNA concentrations were normalized to 20 ng/μL for polymerase chain reaction (PCR).

### 4.2. Identification of LTRs

Our research group completed the whole-genome sequencing of diploid *M. albus*, with a genome size of approximately 1.04 Gb (BioProject ID: PRJNA674670). The *M. albus* LTR sequences (LTRs) were identified using the ‘RepeatMasker’ tool on 20 December 2018 (http://www.repeatmasker.org/cgi-bin/WEBRepeatMasker). The ‘cross_match’ search engine was applied, and ‘Rice’ was specified as the DNA source. Other software parameters were run according to the default options. In addition, the LTR primers were designed using DNAMAN Version 6.0 (Lynnon Corporation, San Ramon, CA, USA) (Table S5).

### 4.3. Primer Selection and PCR Conditions

Four accessions of *M. albus* were randomly selected and used to screen 350 LTR-RT markers by PCR amplification. Amplification was conducted in a 10 μL reaction solution that included 1.0 μL of genomic DNA (20 ng/μL), 4.95 μL of 2× reaction mix (500 μM dNTP, 20 mM Tris–HCl, 100 mM KCl, 3 mM MgCl_2_), 2.0 μL of double-distilled water, 1.0 μL of each LTR primer (4 μM each), and 0.05 μL of 2.5 U/μL Golden DNA Polymerase. The PCR program consisted of a pre-denaturation of 3 min at 94 °C, followed by 35 cycles of 94 °C for 30 s, 50–60 °C for 30 s, and 72 °C for 30 s, and finally, an extension cycle of 7 min at 72 °C. The PCR products were separated in 6.0% nondenaturing polyacrylamide gels (400 V, 1.5 h) for visualization under UV Imager Gel Doc XR+ system lights (Bio-Rad, Hengshui, Hebei, China).

### 4.4. Sequencing of PCR Amplification Products

PCR was conducted in a 25.2 μL volume consisting of 10 μL of 2× reaction mix, 0.2 μL of Golden DNA Polymerase, 8.0 μL of ddH_2_O, 3.0 μL of genomic DNA (20 ng/μL), and 2.0 μL of each primer. The PCR products were sent to a commercial company (Shanghai Sangon Biological Engineering Technology, Shanghai, China) for sequencing (ABI 3730 DNA sequencer). Sequence information was used for multiple sequence alignment using DNAMAN.

### 4.5. Data Analysis

The number of amplified bands was recorded to construct a “0, 1” binary matrix. The polymorphism information content (PIC) and expected heterozygosity (He) were calculated as reported previously [45]. BayeScan.V.2.1 software [46] can be used to detect genetic markers under selection using differences in allele frequencies between populations. The default parameters given in the program were used. The POPGENE 32 software was used to calculate the percentage of polymorphic loci (PPL), number of polymorphic loci (NPL), number of observed alleles (N_A_), number of efficient alleles (Ne), Nei’s (1973) diversity index (h), and Shannon’s information index (I) [47]. Analysis of molecular variance (AMOVA) was carried out using the method of Arlequin suite version 3.5 [48]. The UPGMA dendrogram was produced using Free Tree V.9.1.50 software [49]. To assess the reliability, the field repetition count we used was 100. The phylogram was visualized by TreeViewX V.5.0 software [50]. In addition, the Cophenetic correlation test was carried out by using a matrix comparison plot in NTSYSpc.V.2.1. To subdivide the individuals into different subgroups, a Bayesian clustering analysis was carried out in STRUCTURE 2.3 software [51]. Because of the estimated ‘log probability of data’ [LnP(D)] of STRUCTURE overestimating the number of subgroups [52], we used the ad hoc measure K [53] to estimate the number of groups. Values of K to explore were chosen according to [53] and were calculated in Excel tables. It was run for K = 1–11 with the admixture model, and a total of 20 independent runs were set for each K value and for each run.

## 5. Conclusions

In conclusion, 350 pairs of LTR retrotransposon primers for identifying the molecular markers were designed for *M. albus*. Overall, 47 primer pairs showed high polymorphism among 15 *M. albus* accessions, and all of the polymorphic primer pairs showed transferability among 40 accessions of 18 *Melilotus* species. In addition, the origin of the accessions did not have an influence on the genetic structures in the 18 *Melilotus* species. Our results suggest that the markers investigated here will be useful for studying the genetic diversity, population structure, and germplasm of *Melilotus* species.

## Figures and Tables

**Table 1:** Statistics of the type, number of identified, and designed primers of LTR-RTs.

Total Length of LTR-RTs Screened (bp)	No. of LTR-RTs Identified		No. of Primers Designed for Each Type of LTR-RTs	
585,894,349	Ty1/*Copia*	77,935	RBIP	232
	Ty3/*gypsy*	101,240	IRAP	105
	Unknown	1865	ISBP	10
			REMAP	3

RBIP: retrotransposon-based insertion polymorphisms, IRAP: inter-retrotransposon amplified polymorphisms, ISBP: insertion-site-based polymorphisms, REMAP: retrotransposon-microsatellite amplified polymorphisms.

**Table 2:** Primer sequence, allele size range and polymorphism information for 47 LTR retrotransposon loci among 15 *M. albus* accessions.

Primer Code	Type	Primer Sequence	Allele Size Range (kp)	N_A_	He	PIC
25	RBIP	F:GAGAACTGAGAAGAGGGTC R:CTCCACCTTGACTTGAATC	0.2–0.3	5	0.76	0.72
40	RBIP	F:GAAAGGATTCTGAGCGTAG R:ATACTCTCCACCACTGTCA	0.5–0.7	3	0.67	0.6
41	RBIP	F:GAAAGGATTCTGAGCGTAG R:GTAATACTCTCCACCACTGTC	0.4–0.7	5	0.67	0.63
45	IRAP	F:TATGCTTCAACCTGAGGG R:GTTCATTTCTGCTCGCTC	0.3–0.4	4	0.62	0.56
46	RBIP	F:GAAAGTCTAATGCCGAGG R:AATACTCTCCACCACGGT	0.4–0.5	5	0.45	0.43
54	RBIP	F:TCTCAGACATAGAACCCG R:AGTGATGGTAACCCAACC	0.3–0.4	4	0.59	0.53
55	RBIP	F:GTGTCCACAAAGGATTCC R:TCTCCACAAGACCACTTC	0.2–0.4	4	0.43	0.4
62	IRAP	F:ATTTAGTGGCAGCCCTTC R:GACCTTTCTTTCCGCATC	0.3–0.4	3	0.6	0.52
67	RBIP	F:GACAACTTGAACGGACAAAC R:AGGGTAAAGGCTAAGGGAG	0.15–0.2	3	0.57	0.48
68	RBIP	F: GGGACAACTACATAACTTGG R: GCTGCCACTAAATCAGAG	0.4–0.5	3	0.54	0.44
69	RBIP	F: TCACTTACCTATTGCTCTCC R: TGCTTCCTTGACAGTCTTAG	0.15–0.4	3	0.64	0.57
74	RBIP	F:TTCATACCACTCCGAGAG R:GGATGTCCATTAGAGGCT	0.15–0.3	4	0.68	0.61
76	RBIP	F: TGTGTGTGTGTGTCTGTTCT R: AACCTCGTAGTTCGGGTA	0.4–0.5	3	0.51	0.45
78	RBIP	F: CATCCTGAATAGAGTCCCT R: ATCGGTATCCCTTAGCAC	0.2–0.3	3	0.49	0.43
83	RBIP	F: CTGTAGTATTCAAGGGTGG R: GAAGCCATTCTAAGGGTC	0.2–0.4	4	0.58	0.49
93	RBIP	F: CTCCTTGACTGTTGCCATTA R: GGGAAGAAACCCTGGATT	0.2–0.3	3	0.57	0.51
95	RBIP	F: CCTGAAGAAGAATGGTCC R: GTGGTAAGAAGTTGAAGCC	0.15–0.3	4	0.58	0.52
105	RBIP	F: TCTCAACTCCAATGGCAG R: TTCAGAGGCAGAAGCATC	0.2–0.4	4	0.52	0.43
138	IRAP	F: GCATTGTTGTCACAGTCAAG R:GCAAGTTACTCTTCATACCTGG	0.3–0.7	5	0.64	0.59
146	RBIP	F: ATCCCTTCTCTCCTTCCCT R: TCACCTTGATACTTGCCG	0.1–0.2	3	0.57	0.48
148	IRAP	F: GGTGTGGACAGATAGTAAGG R: GAGTTGGTAGGTTGAGTTTG	0.1–0.2	5	0.77	0.73
149	RBIP	F: CTAAATGGAGGGAAGAGAGA R: GTGACAACTTGAGTGCCA	0.2–0.3	3	0.56	0.46
152	IRAP	F: CTTATCTCCCTCAACAAGC R: CTACAGAAATGGCGACTTC	0.1–0.2	4	0.7	0.65
153	IRAP	F: CAGCAACATAACGAGAACG R:CCGAGAGAAATGAGAGAGAAGT	0.2–0.5	4	0.68	0.62
155	RBIP	F: CTTGTTGCGTTAGTGTGC R: AACTGGGATGGTCCGTAT	0.5–0.7	3	0.42	0.38
170	RBIP	F: GTGACGAGAAGAAGAAAGG R:CACAGATTTACCACTGGC	0.2–0.3	3	0.63	0.56
183	RBIP	F: TTACTAATCCCACCACCC R: GACGAAGGAGAAGAGAATG	0.2–0.4	3	0.56	0.47
196	RBIP	F: GATTGTTCCGATTCAGGC R: AGGACTTGCTGGATTTGG	0.3–0.5	5	0.66	0.6
209	RBIP	F: GTCTCACACACAAGATTCC R: GGTGGTTAGGGAGGTTAT	0.15–0.3	5	0.75	0.71
210	RBIP	F: GTCTCACACACAAGATTCC R: GGTGGTTAGGGAGGTTAT	0.15–0.3	5	0.76	0.72
226	RBIP	F: GCTTCAAGTGTGGTGGAT R: AACGCAACCCTTCTCTCT	0.15–0.3	5	0.64	0.59
229	RBIP	F: ATCGGAATGGACTCTACC R: GTGTATGCGTATGTGTGAG	0.15–0.3	7	0.8	0.77
277	RBIP	F: TCAGATGGAGTTGTGAGG R: GAGGCTAAACCCTACGAT	0.2–0.3	4	0.56	0.52
280	RBIP	F: GAACTGTATGTGTCCAAGG R: CCAGGAAGAGAACAAGAC	0.15–0.3	3	0.58	0.51
281	RBIP	F: AGAGGAAGAAGACAACCG R:GTCACAAAGGATGAGGGT	0.15–0.3	4	0.67	0.61
283	RBIP	F: CCCGAATCTAAGGTCAAAGT R: CACGCAAGAAACACATCAC	0.3–0.5	3	0.52	0.41
284	IRAP	F: ATTTGGACCAGGCACACT R: AAGCACTCCGTCATCGTA	0.2–0.4	4	0.63	0.55
286	IRAP	F: CGGATGATACGAAAGTGAG R: GCTTCTGTTGTTAGCCCAT	0.15–0.3	5	0.73	0.69
290	RBIP	F: ACTAAGGTTCCAGGCTGT R: GACTCATCCAACAATCCC	0.3–0.4	3	0.59	0.51
293	RBIP	F: CGGCAAGGTAGAGAGAAGT R: AATGGGCTTTGGAGTAGG	0.15–0.3	4	0.58	0.52
300	IRAP	F: CTCTCACACATACACAAAGG R: ATCTGGAGTTCTGGAAGTC	0.3–0.5	4	0.56	0.51
301	RBIP	F: CAAGCACGGTAAGTTAGC R: CGAGTTCAAGAGCACCTT	0.15–0.4	4	0.49	0.46
302	RBIP	F: AAAGAGGACAAAGACCCG R: TAGTCAACGCACATACGC	0.2–0.5	5	0.5	0.47
324	IRAP	F: GGTATCAGAGCCTGGTTAG R: AAACAGTCCTCAGTTCCTC	0.15–0.3	5	0.65	0.6
336	IRAP	F: GAGGAAGTAGACGCTTATTG R: GTTGGTGGTGTCATTCAC	0.2–0.3	3	0.57	0.51
350	RBIP	F: TCACAGAGTTTGAGTCCC R: GAAGAAGAAGGTGGGTTC	0.2–0.3	4	0.62	0.54
Mean				3.96	0.61	0.54

N_A_: number of alleles, He: expected heterozygosity, PIC: polymorphic information content, RBIP: retrotransposon-based insertion polymorphisms, IRAP: inter-retrotransposon amplified polymorphisms.

**Table 3:** Genetic variability of 18 *Melilotus* species detected by 47 LTR-RT-based markers.

Species	Accessions	NPL	PPL (%)	N_A_	Ne	h	I
*M. albus*	3	79	27.05	1.2705	1.1679	0.0998	0.1493
*M. altissimus*	2	29	9.93	1.0993	1.0702	0.0411	0.0601
*M. dentatus*	3	50	17.12	1.1712	1.1128	0.0655	0.0970
*M. elegans*	2	45	15.41	1.1541	1.1090	0.0638	0.0932
*M. hirsutus*	2	43	14.73	1.1473	1.1041	0.0610	0.0891
*M. indicus*	3	70	23.97	1.2397	1.1583	0.0918	0.1360
*M. infestus*	3	61	20.89	1.2089	1.1452	0.0826	0.1213
*M. italicus*	3	98	33.56	1.3356	1.2282	0.1309	0.1929
*M. officinalis*	3	49	16.78	1.1678	1.1150	0.0658	0.0968
*M. polonicus*	2	24	8.22	1.0822	1.0581	0.0340	0.0497
*M. segetalis*	1	0	0.00	1	1	0	0
*M. siculus*	3	56	19.18	1.1918	1.1342	0.0762	0.1117
*M. speciosus*	2	38	13.01	1.1301	1.0920	0.0539	0.0787
*M. spicatus*	2	2	0.68	1.0068	1.0048	0.0028	0.0041
*M. suaveolens*	2	17	5.82	1.0582	1.0412	0.0241	0.0352
*M. sulcatus*	1	0	0.00	1	1	0	0
*M. tauricus*	2	17	5.82	1.0582	1.0412	0.0241	0.0352
*M. wolgicus*	1	0	0.00	1	1	0	0
Mean		37.67	12.90	1.1290	1.0879	0.0510	0.0750

NPL: number of polymorphic loci, PPL: the percentage of polymorphic loci, N_A_: observed number of alleles, Ne: effective number of alleles, h: Nei’s (1973) gene diversity, I: Shannon’s information index.

**Table 6:** Accessions of 18 *Melilotus* species used for analysis of primer transferability.

Code	Species	Accession Number	Origin	Latitude	Longitude
1	*M. albus*	PI 342739	England, United Kingdom	N 52°26′	W 19°06′
		PI 595327	China	N 43°18′	E 86°40′
		PI 662299	Vienna, Austria	N 48°20′	E 16°33′
2	*M. altissimus*	Ames 18376	Nebraska, United States	N 41°26′	W 99°23′
		PI 420163	France	N 46°13′	E 2°12′
3	*M. dentatus*	PI 108656	Armenia	N 40°4′	E 45°2′
		PI 90753	China	N 35°51′	E 104°11′
		PI 595334	China	N 42°49′	E 85°30′
4	*M. elegans*	PI 260271	Ethiopia	N 9°9′	E 37°48′
		PI 250873	Iran	N32°4′	E 54°4′
5	*M. hirsutus*	Ames 22882	Russian Federation	—	—
		PI 129697	Sweden	N 60°7′	E 18°38′
6	*M. indicus*	Ames 21619	Nebraska, United States	N 41°29′	W 99°54′
		PI 107562	Uzbekistan	N 41°23′	E 69°4′
		PI 43595	—	—	—
7	*M. infestus*	PI 306326	Algeria	N 27°13′	E 2°29′
		PI 306327	Italy	N 41°52′	E12°34′
		PI 306328	Hungary	N 47°9′	E 19°30′
8	*M. italicus*	PI 317635	Czechoslovakia	N 14°28′	E 121°2′
		PI 317638	Israel	N 31°2′	E 34°51′
		PI 317634	Manitoba Canada	N 56°11′	W 97°4′
9	*M. offcinalis*	PI 132270	Romania	N 45°49′	E 24°29′
		PI 342889	Germany	N 51°13′	E 10°23′
		PI 342898	France	N 46°34′	E 2°18′
10	*M. polonicus*	PI 314386	Former Soviet Union	N 45°5′	E 41°50′
		PI 108647	Former Soviet Union	N 45°5′	E 41°50′
11	*M. segetalis*	PI 317649	Czechoslovakia	N 48°2′	E 18°22′
12	*M. siculus*	PI 129703	Malta	N 35°56′	E 14°22′
		PI 318508	Greece	N 39°4′	E 21°49′
		PI 33366	Former Soviet Union	—	—
13	*M. speciosus*	PI 317650	Manitoba, Canada	N 53°45′	W 98°48′
		PI 64995	Morocco	N 31°52′	W 6°13′
14	*M. spicatus*	Ames 18402	Nebraska, United States	N 41°29′	W 99°54′
		PI 314466	Uzbekistan	—	—
15	*M. suaveolens*	Ames 23793	Mongolia	N 48°10′	E 91°45′
		PI 595395	United States	N 41°52′	W 93°5′
16	*M. sulcatus*	PI 227595	Tunisia	N 33°53′	E 9°32′
17	*M. tauricus*	PI 67510	Ukraine	N 44°24′	E 33°49′
		Ames 25789	Ukraine	N 44°24′	E 33°49′
18	*M. wolgicus*	PI 317666	Czechoslovakia	N 48°2′	E 18°22′

—: note unknown.

## Data Availability

The whole-genome sequencing of diploid *M. albus* was deposited in NCBI (PRJNA674670).

## Supplementary Materials

The following are available online at https://www.mdpi.com/article/10.3390/plants10050890/s1, Table S1: The information of being identified LTR-RTs in the *Melilotus* genome. Table S2: Primer sequence, allele size range and polymorphism information for 47 LTR retrotransposon loci among 18 *Melilotus* species. Table S3: Detection of outlier loci using BayeScan. Table S4: List of *M. albus* accessions used for LTR-RT molecular marker validation. Table S5: Information of LTR primers designed using DNAMAN software. Figure S1: Distribution of the ratio of LTR retrotransposons on eight chromosomes of *Melilotus*. Figure S2: The result of PAGE (polyacrylamide gel electrophoresis) of primer 25 in 15 *M. albus* accessions. Figure S3: Matrix comparison plot for cophenetic correlation test. Figure S4: Dendrogram of 15 *M. albus* by the UPGMA cluster analysis based on LTR-RT analysis.
