# Supplementary material for: Genome-Wide Identification and Development of LTR Retrotransposon-Based Molecular Markers for the Melilotus Genus
Source: Plants (Basel). 2021 Apr 28;10(5):890. doi: 10.3390/plants10050890 (PMC8146837; doi:10.3390/plants10050890)
Supplement: Supplementary file 1 [file plants-10-00890-s001.zip › Supplementary/Table S2-2.pdf]

Primer code	Type	Primer sequence	Allele size range (kb)	N_A	He	PIC
25	RBIP	F:GAGAACTGAGAAGAGGGTC<br>R:CTCCACCTTGACTTGAATC	0.2-0.4	9	0.83	0.81
40	RBIP	F:GAAAGGATTCTGAGCGTAG<br>R:ATACTCTCCACCACTGTCA	0.5-1.0	5	0.63	0.56
41	RBIP	F:GAAAGGATTCTGAGCGTAG<br>R:GTAATACTCTCCACCACTGTC	0.5-1.0	8	0.83	0.81
45	IRAP	F:TATGCTTCAACCTGAGGG<br>R:GTTCATTTCTGCTCGCTC	0.3-0.7	10	0.7	0.65
46	RBIP	F:GAAAGTCTAATGCCGAGG<br>R:AATACTCTCCACCACGGT	0.2-0.7	9	0.7	0.65
54	RBIP	F:TCTCAGACATAGAACCCG<br>R:AGTGATGGTAACCCAACC	0.3-0.7	10	0.79	0.76
55	RBIP	F:GTGTCCACAAAGGATTCC<br>R:TCTCCACAAGACCACTTC	0.15-0.4	8	0.73	0.69
62	IRAP	F:ATTTAGTGGCAGCCCTTC<br>R:GACCTTTCTTTCCGCATC	0.1-1.0	15	0.84	0.82
67	RBIP	F:GACAACTTGAACGGACAAAC<br>R:AGGGTAAAGGCTAAGGGAG	0.15-0.3	6	0.73	0.68
68	RBIP	F:GGGACAACTACATAACTTGG<br>R:GCTGCCACTAAATCAGAG	0.3-0.7	11	0.83	0.81
69	RBIP	F:TCACTTACCTATTGCTCTCC<br>R:TGCTTCCTTGACAGTCTTAG	0.15-0.3	6	0.69	0.66
74	RBIP	F:TTCATACCACTCCGAGAG<br>R:GGATGTCCATTAGAGGCT	0.15-0.2	4	0.43	0.4
76	RBIP	F:TGTGTGTGTGTGTCTGTTCT<br>R:AACCTCGTAGTTCGGGTA	0.3-0.5	5	0.78	0.74
78	RBIP	F:CATCCTGAATAGAGTCCCT<br>R:ATCGGTATCCCTTAGCAC	0.15-0.3	4	0.57	0.52
83	RBIP	F:CTGTAGTATTCAAGGGTGG<br>R:GAAGCCATTCTAAGGGTC	0.2-0.5	7	0.56	0.54
93	RBIP	F:CTCCTTGACTGTTGCCATTA<br>R:GGGAAGAAACCCTGGATT	0.2-0.4	5	0.37	0.35
95	RBIP	F:CCTGAAGAAGAATGGTCC<br>R:GTGGTAAGAAGTTGAAGCC	0.1-0.3	8	0.81	0.78
105	RBIP	F:TCTCAACTCCAATGGCAG<br>R:TTCAGAGGCAGAAGCATC	0.15-0.3	7	0.8	0.77
138	IRAP	F:GCATTGTTGTCACAGTCAAG<br>R:GCAAGTTACTCTTCATACCTGG	0.5-1.0	4	0.67	0.61
146	RBIP	F:ATCCCTTCTCTCCTTCCCT<br>R:TCACCTTGATACTTGCCG	0.15-0.3	5	0.78	0.75
148	IRAP	F:GGTGTGGACAGATAGTAAGG	0.15-0.3	4	0.71	0.66

		R:GAGTTGGTAGGTTGAGTTTG				
149	RBIP	F:CTAAATGGAGGGAAGAGAGA<br>R:GTGACAACTTGAGTGCCA	0.2-0.3	5	0.74	0.7
152	IRAP	F:CTTATCTCCCTCAACAAGC<br>R:CTACAGAAATGGCGACTTC	0.15-0.2	3	0.61	0.53
153	IRAP	F:CAGCAACATAACGAGAACG<br>R:CCGAGAGAAATGAGAGAGAAGT	0.4-0.7	8	0.77	0.75
155	RBIP	F:CTTGTTGCGTTAGTGTGC<br>R:AACTGGGATGGTCCGTAT	0.5-1.0	6	0.72	0.68
169	IRAP	F:GTGAATGTGTGTGTGTGC<br>R:CTTTGAGCAAGTGTGTAGGT	0.3-0.5	7	0.83	0.81
170	RBIP	F:GTGACGAGAAGAAGAAAGG<br>R:CACAGATTTACCACTGGC	0.2-0.3	4	0.69	0.63
183	RBIP	F:TTACTAATCCCACCACCC<br>R:GACGAAGGAGAAGAGAATG	0.3-0.5	5	0.73	0.68
196	RBIP	F:GATTGTTCCGATTCAGGC<br>R:AGGACTTGCTGGATTTGG	0.2-0.4	6	0.78	0.75
209	RBIP	F:GTCTCACACACAAGATTCC<br>R:GGTGGTTAGGGAGGTTAT	0.15-0.4	7	0.84	0.82
210	RBIP	F:GTCTCACACACAAGATTCC<br>R:GGTGGTTAGGGAGGTTAT	0.15-0.3	6	0.72	0.67
226	RBIP	F:GCTTCAAGTGTGGTGGAT<br>R:AACGCAACCCTTCTCTCT	0.2-0.3	5	0.68	0.63
229	RBIP	F:ATCGGAATGGACTCTACC<br>R:GTGTATGCGTATGTGTGAG	0.1-0.3	10	0.83	0.81
277	RBIP	F:TCAGATGGAGTTGTGAGG<br>R:GAGGCTAAACCCTACGAT	0.2-0.3	3	0.62	0.54
280	RBIP	F:GAACTGTATGTGTCCAAGG<br>R:CCAGGAAGAGAACAAGAC	0.2-0.3	4	0.68	0.63
281	RBIP	F:AGAGGAAGAAGACAACCG<br>R:GTCACAAAGGATGAGGGT	0.15-0.3	4	0.45	0.42
283	IRAP	F:CCCGAATCTAAGGTCAAAGT<br>R:CACGCAAGAAACACATCAC	0.15-0.5	10	0.86	0.84
284	IRAP	F:ATTTGGACCAGGCACACT<br>R:AAGCACTCCGTCATCGTA	0.1-0.3	6	0.7	0.65
286	IRAP	F:CGGATGATACGAAAGTGAG<br>R:GCTTCTGTTGTTAGCCCAT	0.3-0.5	4	0.66	0.6
290	RBIP	F:ACTAAGGTTCCAGGCTGT<br>R:GACTCATCCAACAATCCC	0.2-0.4	3	0.4	0.37
293	RBIP	F:CGGCAAGGTAGAGAGAAGT<br>R:AATGGGCTTTGGAGTAGG	0.1-0.3	8	0.79	0.76
300	IRAP	F:CTCTCACACATACACAAAGG<br>R:ATCTGGAGTTCTGGAAGTC	0.2-0.5	5	0.7	0.66
301	RBIP	F:CAAGCACGGTAAGTTAGC	0.2-0.4	4	0.68	0.62

		R:CGAGTTCAAGAGCACCTT				
302	RBIP	F:AAAGAGGACAAAGACCCG<br>R: TAGTCAACGCACATACGC	0.3-0.5	3	0.5	0.38
324	IRAP	F:GGTATCAGAGCCTGGTTAG<br>R:AAACAGTCCTCAGTTCCTC	0.2-0.3	4	0.56	0.5
336	IRAP	F:GAGGAAGTAGACGCTTATTG<br>R:GTTGGTGGTGTCATTCAC	0.2-0.4	5	0.76	0.72
350	RBIP	F:TCACAGAGTTTGAGTCCC<br>R:GAAGAAGAAGGTGGGTTC	0.15-0.4	7	0.84	0.82
Mean				6.21	0.70	0.66

N_A: number of alleles, He: expected heterozygosity, PIC: polymorphic information content, RBIP: retrotransposon-based insertion polymorphisms, IRAP: inter-retrotransposon amplified polymorphisms.
