# Supplementary material for: Genome-Wide Identification and Development of LTR Retrotransposon-Based Molecular Markers for the Melilotus Genus
Source: Plants (Basel). 2021 Apr 28;10(5):890. doi: 10.3390/plants10050890 (PMC8146837; doi:10.3390/plants10050890)
Supplement: Supplementary file 1 [file plants-10-00890-s001.zip › Supplementary/Table S2.pdf]

Code	Accession number	Origin	Latitude	Longitude
1	PI 662299	Vienna, Austria	N 48°20′	E 16°33′
2	PI 553075	Poland	N 51°48′	E 19°06′
3	PI 366038	Buenos Aires, Argentina	S 34°35′	W 58°26′
4	PI 478773	Florida, United States	N 27°39′	W 81°30′
5	PI 508617	Santa Fe	S 32°5′	E 1°29′
6	PI 342796	Hungary	N 47°05′	E 19°36′
7	PI 662296	Saskatchewan, Canada	—	—
8	PI 478468	Bolivia	—	—
9	PI 342765	France	N 46°15′	W 2°16′
10	PI 494706	Romania	N 44°12′	E 28°36′
11	ZXY06P-1732	Russian Federation	N 62°	W 9°
12	Zhongxu-1226	—	—	—
13	ZXY07P-3150	—	—	—
14	ZXY05P-983	Russian Federation	—	—
15	HB2009-153	Xinyang, China	N 32°10′	E 114°07′
