# Supplementary material for: Genome-Wide Identification and Development of LTR Retrotransposon-Based Molecular Markers for the Melilotus Genus
Source: Plants (Basel). 2021 Apr 28;10(5):890. doi: 10.3390/plants10050890 (PMC8146837; doi:10.3390/plants10050890)
Supplement: Supplementary file 1 [file plants-10-00890-s001.zip › Supplementary/Table S4.pdf]

Marker name	TE ID	Type	Forward primer (5'-3')	Reverse primer (5'-3')	Class	Chromosome
Ma_LTR_1	repeat_TE0090401	RBIP	CCATTGATTGAGGCAGTAG	CAAGTCTATGTTCCAAACCC	*Copia*	Group3
Ma_LTR_2	repeat_TE0090415	RBIP	TTGTTCAGGAGTTCCAGG	CACATAACCTTTCTTCCACC	*Copia*	Group3
Ma_LTR_3	repeat_TE0090644	RBIP	CACATAACCTTGCTTCCG	CGTAATACTCTCCACCACG	*Copia*	Group3
Ma_LTR_4	repeat_TE0090728	RBIP	GTTCCCAGAAGTTGAGACT	GCAATAAAGTGACCAGGC	*Copia*	Group3
Ma_LTR_5	repeat_TE0090729	RBIP	GATTGATACTCCTGAACCTC	TCCTTCCTTGTAGACACAC	*Copia*	Group3
Ma_LTR_6	repeat_TE0090729	IRAP	GATGACAAAGAGCCTGAC	CATTGAGATGAGACCAAGC	*Copia*	Group3
Ma_LTR_7	repeat_TE0090866	ISBP	CCCACAACTCCTCAACATT	CACACAAAGCCTATTTCAGG	*Copia*	Group3
Ma_LTR_8	repeat_TE0128816	RBIP	CTTATTCCCATCTTGCCC	CCTCTTGTGAAAGTCGTTTG	*Copia*	Lachesis_group3:72975954-72981950
Ma_LTR_9	repeat_TE0128835	RBIP	CAAGTTGTATGGGTATCCTC	GCGTAGAGTAACCACTGTC	*Copia*	Lachesis_group3:72994466-72996400
Ma_LTR_10	repeat_TE0128985	IRAP	CCCATTGATTGAGGCAGT	TCTATGTTCCAAACCCTGAG	*Copia*	Lachesis_group3:73054159-73063470
Ma_LTR_11	repeat_TE0128987	RBIP	AGCCACTCTAAAGGTCTG	GTCTATGTTCCAAACCCTG	*Copia*	Lachesis_group3:73063736-73070466
Ma_LTR_12	repeat_TE0128991	IRAP	GTTCGGAGCATTAGAAGC	CCAACTTGTTTACAGCCC	*Copia*	Lachesis_group3:73071984-73073433
Ma_LTR_13	repeat_TE0128992	IRAP	GTTCGGAGCATTAGAAGC	GAGCCAACTTGTTTACAGC	*Copia*	Lachesis_group3:73072235-73074083
Ma_LTR_14	repeat_TE0129038	IRAP	GCTTCCGTCCTTCTATGA	GACCAAGTGTATCGCCTT	*Copia*	Lachesis_group3:73099627-73100311
Ma_LTR_15	repeat_TE0140174	RBIP	AGATTTCTCACTCTCAGGG	GTTGGTTACACTTCTAAGGC	*Copia*	Lachesis_group3:81546882-81547720
Ma_LTR_16	repeat_TE0140260	RBIP	GGCTTATCAATGGAGTCAAC	GACAGGAAATCAACTTCTGG	*Copia*	Lachesis_group3:81571424-81573046
Ma_LTR_17	repeat_TE0140261	IRAP	GAAGAGGCAACAAGAGTTTG	AAGAAGTGATGAGGGCAC	*Copia*	Lachesis_group3:81573045-81574425

Ma_LTR_18	repeat_TE0140262	IRAP	CACTGCTTCGTGATACCA	CCCTTTACATCCTCAACAG	*Copia*	Lachesis_group3:81573869-81576068
Ma_LTR_19	repeat_TE0140548	ISBP	GGGACAACTACATAACTTGG	GCTGCCACTAAATCAGAG	*Copia*	Lachesis_group3:81703352-81710186
Ma_LTR_20	repeat_TE0165557	RBIP	ATGATAGATGGAGCCGTC	GATGTCTTTCTTGAGCAGC	*Copia*	Lachesis_group3:96037227-96040455
Ma_LTR_21	repeat_TE0165557	REMAP	TCACATCACGAGTATGGG	TTAGCCAAAGAGGACGAG	*Copia*	Lachesis_group3:96037227-96040455
Ma_LTR_22	repeat_TE0165599	RBIP	CAATGTGAGTCCTAATCTGG	ATGGGAATGGTGAGAGGT	*Copia*	Lachesis_group3:96052163-96056458
Ma_LTR_23	repeat_TE0165599	RBIP	CACCTTCTTCAAGTAACCAG	AGGAGGAGATTGGAAAGC	*Copia*	Lachesis_group3:96052163-96056458
Ma_LTR_24	repeat_TE0165599	IRAP	CCCTATCACGGTTTCTAATG	CTGGTATTGACTTGAAGACACC	*Copia*	Lachesis_group3:96052163-96056458
Ma_LTR_25	repeat_TE0240683	RBIP	GAGAACTGAGAAGAGGGTC	CTCCACCTTGACTTGAATC	*Copia*	Lachesis_group4:13608775-13610337
Ma_LTR_26	repeat_TE0240741	RBIP	CAATCTCCACCTTGACTCT	TGGCTCTATCTACCACTCA	*Copia*	Lachesis_group4:13649291-13654820
Ma_LTR_27	repeat_TE0240782	IRAP	CAGGAACATCACACAGGA	CTTGGAAGACCTTGACAAC	*Copia*	Lachesis_group4:13667161-13668148
Ma_LTR_28	repeat_TE0240782	RBIP	GACACTAATGGCTTGGAC	ACCTTGGAAGACCTTGAC	*Copia*	Lachesis_group4:13667161-13668148
Ma_LTR_29	repeat_TE0240956	RBIP	GGAAGTCAAAGAAGCAGG	ACACCATCACTCCTCCTTG	*Copia*	Lachesis_group4:13756592-13761111
Ma_LTR_30	repeat_TE0241043	RBIP	CAAGTATTGGAGGCTTCC	GGACGGGTTTGAGTATGA	*Copia*	Lachesis_group4:13822302-13827612
Ma_LTR_31	repeat_TE0241043	RBIP	AGCAGAGGACCGACTAACA	TGACATCATCACCCGTTC	*Copia*	Lachesis_group4:13822302-13827612
Ma_LTR_32	repeat_TE0241043	IRAP	AGTTGCTTGAGATGGCTG	CACTCTTGGCATTGTAACC	*Copia*	Lachesis_group4:13822302-13827612
Ma_LTR_33	repeat_TE0241068	RBIP	GTCAGTTAGGTCGGGTTA	CACTATGGAATCTGGGATG	*Copia*	Lachesis_group4:13834901-13842555
Ma_LTR_34	repeat_TE0241730	RBIP	CGGACAAATGTGATAAGAGC	CGGATGTAACAAGACCGA	*Copia*	Lachesis_group4:14110161-14114831
Ma_LTR_35	repeat_TE0241730	RBIP	CTTGTGTCAAATGAGATGGC	ATTGGGTCTGAACTGGGT	*Copia*	Lachesis_group4:14110161-14114831

Ma_LTR_36	repeat_TE0291411	REMAP	GGTTGATGATGATGAGGAG	GAGACAGATTTAGAGCCG	*Copia*	Lachesis_group4:39070591-39072153
Ma_LTR_37	repeat_TE0291525	IRAP	GGAAGAAAGCCAAGTATCTC	CAATCCATCCACCATCTG	*Copia*	Lachesis_group4:39107485-39110976
Ma_LTR_38	repeat_TE0320379	RBIP	GTAGTGCTTTCTTGAATGGG	CACAATACTCGTAACACGCT	*Copia*	Lachesis_group4:58061857-58066116
Ma_LTR_39	repeat_TE0320389	RBIP	AACGAAACTGACTCCGAG	TGGTAAGGTCTCAACCGT	*Copia*	Lachesis_group4:58072301-58073824
Ma_LTR_40	repeat_TE0320561	RBIP	GAAAGGATTCTGAGCGTAG	ATACTCTCCACCACTGTCA	*Copia*	Lachesis_group4:58150099-58153483
Ma_LTR_41	repeat_TE0320562	RBIP	GAAAGGATTCTGAGCGTAG	GTAATACTCTCCACCACTGTC	*Copia*	Lachesis_group4:58151507-58155892
Ma_LTR_42	repeat_TE0320562	IRAP	TATGCTTCAACCTGAGGG	GTTCATTTCTGCTCGCTC	*Copia*	Lachesis_group4:58151507-58155892
Ma_LTR_43	repeat_TE0320562	RBIP	TGTTATGCTGCCGCTACT	ACAATGGTCCTAAGTCCC	*Copia*	Lachesis_group4:58151507-58155892
Ma_LTR_44	repeat_TE0320563	IRAP	GTTGAGCGAGCAGAAATG	AGTAGCGGCAGCATAACA	*Copia*	Lachesis_group4:58153478-58156630
Ma_LTR_45	repeat_TE0320563	IRAP	TATGCTTCAACCTGAGGG	GTTCATTTCTGCTCGCTC	*Copia*	Lachesis_group4:58153478-58156630
Ma_LTR_46	repeat_TE0438065	RBIP	GAAAGTCTAATGCCGAGG	AATACTCTCCACCACGGT	*Copia*	Lachesis_group5:15775517-15781077
Ma_LTR_47	repeat_TE0438240	RBIP	ACCCACACTTGAGTAGCA	CCCAAATAGTATGGTAGGC	*Copia*	Lachesis_group5:15849444-15851675
Ma_LTR_48	repeat_TE0520883	RBIP	TGATGACTGAACACTGACC	TTGCTATTCCTGGACACC	*Copia*	Lachesis_group5:55296943-55301324
Ma_LTR_49	repeat_TE0520888	RBIP	CTAACGACTTCCCAGCAA	GCCTTTACCAAATCCGAC	*Copia*	Lachesis_group5:55305642-55307992
Ma_LTR_50	repeat_TE0520888	RBIP	CGTTTATTGACTCAGTTGGG	GCTCTTTACTCCTCTCTCACAA	*Copia*	Lachesis_group5:55305642-55307992
Ma_LTR_51	repeat_TE0520988	RBIP	ACCTGATTGCTTCCAGTC	CTGATGTTGCTCATAACGG	*Copia*	Lachesis_group5:55359445-55365106
Ma_LTR_52	repeat_TE0520988	RBIP	GTCCATCTGCTGTAGTCAT	CAACATCTGGTAAGAGGC	*Copia*	Lachesis_group5:55359445-55365106
Ma_LTR_53	repeat_TE0521061	RBIP	TCTCAGACATAGAACCCG	GTGATGGTAACCCAACCT	*Copia*	Lachesis_group5:55423972-55424881

Ma_LTR_54	repeat_TE0521060	RBIP	TCTCAGACATAGAACCCG	AGTGATGGTAACCCAACC	*Copia*	Lachesis_group5:55423842-55424825
Ma_LTR_55	repeat_TE0557485	RBIP	GTGTCCACAAAGGATTCC	TCTCCACAAGACCACTTC	*Copia*	Lachesis_group5:81371650-81374387
Ma_LTR_56	repeat_TE0557485	IRAP	CGTGGGTAGTAATGACAGT	GGTCCAAACTTGAGCATAC	*Copia*	Lachesis_group5:81371650-81374387
Ma_LTR_57	repeat_TE0557575	RBIP	CATCAATGGGCTGACATC	CGTGGGTTTATTAGTGGGA	*Copia*	Lachesis_group5:81406495-81410802
Ma_LTR_58	repeat_TE0557793	RBIP	GTTCAGAAGGGAGTTATGG	GCCCAACAGTTATTACCC	*Copia*	Lachesis_group5:81530745-81536867
Ma_LTR_59	repeat_TE0557810	RBIP	AGGACAGAAGCAACCTGA	CCAGAAGCAACACAATCTC	*Copia*	Lachesis_group5:81564745-81566738
Ma_LTR_60	repeat_TE0634399	RBIP	GGGACAACTACATAACTTGG	GCTGCCACTAAATCAGAG	*Copia*	Lachesis_group5:124483234-124486904
Ma_LTR_61	repeat_TE0634400	RBIP	GAAGGAGACAATCCAGGA	GCTGCCACTAAATCAGAG	*Copia*	Lachesis_group5:124483977-124490204
Ma_LTR_62	repeat_TE0634400	IRAP	ATTTAGTGGCAGCCCTTC	GACCTTTCTTTCCGCATC	*Copia*	Lachesis_group5:124483977-124490204
Ma_LTR_63	repeat_TE0652500	RBIP	GATGAAGATGTCAATGCGAC	GGCAATGTGGATACGAGA	*Copia*	Lachesis_group5:132351735-132353229
Ma_LTR_64	repeat_TE0652501	RBIP	GATGAAGATGTCAATGCGAC	GGCAATGTGGATACGAGA	*Copia*	Lachesis_group5:132351946-132355938
Ma_LTR_65	repeat_TE0652501	IRAP	AATGATGCTCGCCACTAC	GTGAGGTATGTTCCCGAA	*Copia*	Lachesis_group5:132351946-132355938
Ma_LTR_66	repeat_TE0652756	RBIP	CAATCTCCACCTTGACTC	TGGCTCTATCTACCACTCA	*Copia*	Lachesis_group6:15781-17997
Ma_LTR_67	repeat_TE0652758	RBIP	GACAACTTGAACGGACAAAC	AGGGTAAAGGCTAAGGGAG	*Copia*	Lachesis_group6:17998-20199
Ma_LTR_68	repeat_TE0653117	RBIP	GGGACAACTACATAACTTGG	GCTGCCACTAAATCAGAG	*Copia*	Lachesis_group6:185622-192348
Ma_LTR_69	repeat_TE0652809	RBIP	TCACTTACCTATTGCTCTCC	TGCTTCCTTGACAGTCTTAG	*Copia*	Lachesis_group6:51419-53605
Ma_LTR_70	repeat_TE0652869	RBIP	GCACATAAGATGGACACC	CTCTAACTGAAAGGGCAAG	*Copia*	Lachesis_group6:75607-76233
Ma_LTR_71	repeat_TE0711137	RBIP	GCAGACATACGGTAAGGT	TAAGGGTTCGGTGACTAC	*Copia*	Lachesis_group6:31344216-31348042

Ma_LTR_72	repeat_TE0711177	RBIP	GGCATCAGAGCAAGTTATG	GTTGTAGGAATGAATGGACC	*Copia*	Lachesis_group6:31372611-31375702
Ma_LTR_73	repeat_TE0711226	RBIP	GCTTCTTGGTTGACTCAG	GACAATACTGCTGCCATC	*Copia*	Lachesis_group6:31442137-31443294
Ma_LTR_74	repeat_TE0723322	RBIP	TTCATACCACTCCGAGAG	GGATGTCCATTAGAGGCT	*Copia*	Lachesis_group6:40405125-40410808
Ma_LTR_75	repeat_TE0723332	RBIP	TTCCACTCAGAAGCAAGC	TTGTCAGTTCCTGGGAGAG	*Copia*	Lachesis_group6:40414809-40421702
Ma_LTR_76	repeat_TE0723336	RBIP	TGTGTGTGTGTCTGTTCT	AACCTCGTAGTTCGGGTA	*Copia*	Lachesis_group6:40422912-40426002
Ma_LTR_77	repeat_TE0723763	IRAP	GTTGTCCAGCAGAGGTAAA	CCCATACATTATTCACCGAC	*Copia*	Lachesis_group6:40607978-40615096
Ma_LTR_78	repeat_TE0723763	RBIP	CATCCTGAATAGAGTCCCT	ATCGGTATCCCTTAGCAC	*Copia*	Lachesis_group6:40607978-40615096
Ma_LTR_79	repeat_TE0726001	RBIP	GTCTTGAATAGTGTGAGCC	TGAAGGGAGTCAGGTCTA	*Copia*	Lachesis_group6:42034875-42040993
Ma_LTR_80	repeat_TE0726002	RBIP	GAAGTCCCATTTCATCTGC	AACTCATCCAGTGCCTTG	*Copia*	Lachesis_group6:42040994-42042759
Ma_LTR_81	repeat_TE0726019	RBIP	TCTTTAGACCTGACTCCCT	CACATCTCGCCTTACTACA	*Copia*	Lachesis_group6:42048081-42055965
Ma_LTR_82	repeat_TE0726019	RBIP	CTCACCTGTATCTTTGCC	GTGTAATGGAGAAGGACG	*Copia*	Lachesis_group6:42048081-42055965
Ma_LTR_83	repeat_TE0726155	RBIP	CTGTAGTATTCAAGGGTGG	GAAGCCATTCTAAGGGTC	*Copia*	Lachesis_group6:42120797-42128815
Ma_LTR_84	repeat_TE0726166	RBIP	CCCAGAGTTTGAACCATAG	GCAGAAGCAGAATACATCTC	*Copia*	Lachesis_group6:42136410-42139831
Ma_LTR_85	repeat_TE0754326	RBIP	GGGTTGTGGAAGGTAAAG	CCTATGTGCCAAGGACTT	*Copia*	Lachesis_group0:7173588-7176491
Ma_LTR_86	repeat_TE0754326	IRAP	TGAAGCAACAACTCTACTCC	ATCCTCTACATCATCCGC	*Copia*	Lachesis_group0:7173588-7176491
Ma_LTR_87	repeat_TE0754487	RBIP	ATAGAGGCTGAACACGCT	ATCGGAAGAGAATCTGGC	*Copia*	Lachesis_group0:7225114-7229272
Ma_LTR_88	repeat_TE0754488	RBIP	AGTATCCCGAGTCCAAGAC	CAAGATGAAGATGTGAGCAC	*Copia*	Lachesis_group0:7226013-7230587
Ma_LTR_89	repeat_TE0755070	RBIP	CTGTAACCTTCAACTACCAGTC	CCACGAAACCAGTCATCTA	*Copia*	Lachesis_group0:7430142-7431963

Ma_LTR_90	repeat_TE0755797	RBIP	CAGTTTGACGAACGCTCTA	CAATAGGATGGGACTAATGC	*Copia*	Lachesis_group0:7649419-7655630
Ma_LTR_91	repeat_TE0764449	RBIP	GGACCAATGTCAACGAAAG	ACTGGAACAAAGGCAAGC	*Copia*	Lachesis_group0:11289587-11293702
Ma_LTR_92	repeat_TE0764449	IRAP	TGAGGGACCAATGTCAAC	GAGCAAAGGTTATCACGAC	*Copia*	Lachesis_group0:11289587-11293702
Ma_LTR_93	repeat_TE0764465	RBIP	CTCCTTGACTGTTGCCATTA	GGGAAGAAACCCTGGATT	*Copia*	Lachesis_group0:11302117-11303365
Ma_LTR_94	repeat_TE0764552	RBIP	GTTGGCTTTATCTACCACTC	CAATCTCCACCTTGACTC	*Copia*	Lachesis_group0:11330898-11333322
Ma_LTR_95	repeat_TE0765051	RBIP	CCTGAAGAAGAATGGTCC	GTGGTAAGAAGTTGAAGCC	*Copia*	Lachesis_group0:11547290-11547955
Ma_LTR_96	repeat_TE0785042	RBIP	GATGACAAAGAGCCTGAC	GAGTTTCTGGATGAGCAG	*Copia*	Lachesis_group0:20534578-20541377
Ma_LTR_97	repeat_TE0785043	RBIP	AGAGACTATGTTCAGAAGGG	CTTTCCCACTTATGGACAC	*Copia*	Lachesis_group0:20538853-20544590
Ma_LTR_98	repeat_TE0785081	RBIP	GGTTTGACAACTGGGCTA	CAACACAACAACAGATGAGC	*Copia*	Lachesis_group0:20560489-20565285
Ma_LTR_99	repeat_TE0785081	RBIP	ATGTGAGAAGTGGCTCCA	AACTACTACAACGGCGGA	*Copia*	Lachesis_group0:20560489-20565285
Ma_LTR_100	repeat_TE0785428	RBIP	TGCCTCTCCTACATAACAAG	GCTACCCAAGTGACAAGAA	*Copia*	Lachesis_group0:20669192-20670287
Ma_LTR_101	repeat_TE0785465	RBIP	GCCTAAGTCCCTTGGTTTA	TGTTATGCTGCCGCTACT	*Copia*	Lachesis_group0:20679015-20685786
Ma_LTR_102	repeat_TE0785482	RBIP	AAGAGGAAGAAGTGAGCAC	ATAAGAGCATCCCGAGTC	*Copia*	Lachesis_group0:20691015-20696606
Ma_LTR_103	repeat_TE0785694	RBIP	CCAGTCAAATGGAGACCTA	GCTCTTCTTTCTTAGCACC	*Copia*	Lachesis_group0:20765871-20769425
Ma_LTR_104	repeat_TE0785696	IRAP	GGATGACCATAAAGTCGG	CCTGTAGGTTCTCGTAACG	*Copia*	Lachesis_group0:20769422-20770889
Ma_LTR_105	repeat_TE0785723	RBIP	TCTCAACTCCAATGGCAG	TTCAGAGGCAGAAGCATC	*Copia*	Lachesis_group0:20804730-20809941
Ma_LTR_106	repeat_TE1457294	REMAP	CGGTATTCACATAACTCCCT	TCCTCGGTAGGTAATCCA	*Copia*	Lachesis_group1:125707-127979
Ma_LTR_107	repeat_TE1457489	RBIP	CACACTCATCACTGCCTA	AGACTATGTTCAGAAGGGAG	*Copia*	Lachesis_group1:179017-185339

Ma_LTR_108	repeat_TE1457494	RBIP	GCTTTGTCCTTCCTTGTAGA	ACCTGAGAGTTCATCTGTCC	*Copia*	Lachesis_group1:203179-208199
Ma_LTR_109	repeat_TE1457502	IRAP	GGTCTTGATAAGTTGAAGGC	GGACACAGAGGAAATAGCA	*Copia*	Lachesis_group1:212363-215374
Ma_LTR_110	repeat_TE1457502	IRAP	CCCATTGACACACCTATG	TTCCTCTTCCTGGATACC	*Copia*	Lachesis_group1:212363-215374
Ma_LTR_111	repeat_TE1457588	RBIP	CCCACTAACCAAGCACAT	GACAGGAAATCAACTTCTGG	*Copia*	Lachesis_group1:235096-239160
Ma_LTR_112	repeat_TE1457588	RBIP	ATACTCTCCACCACGGTCA	TGTTCACCTTGTCATCCG	*Copia*	Lachesis_group1:235096-239160
Ma_LTR_113	repeat_TE1482138	RBIP	TGCTTCCAGTCTTGCTAC	GCACAACTTGATGTCACTC	*Copia*	Lachesis_group1:14199634-14206773
Ma_LTR_114	repeat_TE1482256	RBIP	AGAGAGACTGCCTGTTACA	GGAGGATAGAAGTGTGACA	*Copia*	Lachesis_group1:14320335-14322364
Ma_LTR_115	repeat_TE1482257	RBIP	CAAGCAGCAAGAGAGGTT	AGGAAGCCAAAGGTTAGC	*Copia*	Lachesis_group1:14321555-14327337
Ma_LTR_116	repeat_TE1482258	RBIP	CCTCCCAAAGAGAGAGAGT	TACGGTGTGTGTGAAAGC	*Copia*	Lachesis_group1:14327340-14328598
Ma_LTR_117	repeat_TE1513724	RBIP	AGATGTTGTCTGTGGAGC	CTGGGAGAAGAGAACTCA	*Copia*	Lachesis_group1:34602168-34602951
Ma_LTR_118	repeat_TE1513829	RBIP	CATTCAGACTCCATTTGTGG	GTCCATACTTCTTCCCATTG	*Copia*	Lachesis_group1:34693263-34695817
Ma_LTR_119	repeat_TE1528954	RBIP	TCAATGTGAAGCCACTCC	ATGTTCCAAACCCTGAGC	*Copia*	Lachesis_group1:44915054-44923260
Ma_LTR_120	repeat_TE1529254	IRAP	GGAAATGGAGGATGACCA	GACGATTGAAGTTGCTAAGG	*Copia*	Lachesis_group1:45046506-45048235
Ma_LTR_121	repeat_TE1529335	RBIP	CCAACCTAAATGACTGGAG	CACACTCAATCTTTCAGGG	*Copia*	Lachesis_group1:45085559-45088781
Ma_LTR_122	repeat_TE1529380	RBIP	AGGCTTAGAGAGAGATGGG	GACATTCTTGTGACTTGGG	*Copia*	Lachesis_group1:45132620-45137757
Ma_LTR_123	repeat_TE1529380	RBIP	AGTTCCTACGGCTAATGC	GCGGTAAGACTGGAGATT	*Copia*	Lachesis_group1:45132620-45137757
Ma_LTR_124	repeat_TE1529403	IRAP	CCCAAGTCACAAGAATGTC	ACAGGAGAGATGTCTAAGGG	*Copia*	Lachesis_group1:45147393-45151384
Ma_LTR_125	repeat_TE1697693	RBIP	CACAGTCCCAGTGATTTAC	GAGAGAGATTGAAGCCAC	*Copia*	Lachesis_group2:1009621-1010361

Ma_LTR_126	repeat_TE1697693	IRAP	CAAACAATCACAGTCCCAGT	TTCCAACATCTGAGAGCG	*Copia*	Lachesis_group2:1009621-1010361
Ma_LTR_127	repeat_TE1697941	RBIP	TAAAGTCGCCACCGAAGT	GGTTCCCGTTGGATACAA	*Copia*	Lachesis_group2:1076677-1077292
Ma_LTR_128	repeat_TE1697944	RBIP	CACAAACTCCACCACCAA	GAAATCCGAGGAATCTTGAC	*Copia*	Lachesis_group2:1094908-1095610
Ma_LTR_129	repeat_TE1697945	IRAP	CATCTTTACCTTCCACAACC	GGTGAACAATCTCTTGGTG	*Copia*	Lachesis_group2:1094992-1096797
Ma_LTR_130	repeat_TE1698337	RBIP	CTCCCACTTCTTCTCTTATG	GTTCACCACTTTCCTCACT	*Copia*	Lachesis_group2:1233831-1236424
Ma_LTR_131	repeat_TE1699394	IRAP	AAGTGGTGAACCTTAGGC	GATGTCCCATTTAGAACCC	*Copia*	Lachesis_group2:1652124-1655724
Ma_LTR_132	repeat_TE1699394	RBIP	GTGGGAAACTTGGATTGG	CAAACTTTACTCGGTTGCC	*Copia*	Lachesis_group2:1652124-1655724
Ma_LTR_133	repeat_TE1699395	ISBP	AAGGATGGTAGAGAGCAGC	TTCACTCACCCAAACCCT	*Copia*	Lachesis_group2:1655725-1657732
Ma_LTR_134	repeat_TE1699603	RBIP	GATGTCCCATTTAGAACCC	CTACCTTATTTGCTCCACAC	*Copia*	Lachesis_group2:1717345-1722264
Ma_LTR_135	repeat_TE1699603	IRAP	CTCCGAGACATAGCACAA	GGAAAGTGGTGAACCTTAG	*Copia*	Lachesis_group2:1717345-1722264
Ma_LTR_136	repeat_TE1699697	IRAP	CCCACTTTGAACCTATGG	TCTTCTCTCTCTCTCGCTG	*Copia*	Lachesis_group2:1751319-1754074
Ma_LTR_137	repeat_TE1697674	RBIP	CAGTCTGAGCAACCTTCA	CTTCATCATCTTCCTGAGC	*Copia*	Lachesis_group2:990918-992537
Ma_LTR_138	repeat_TE1697674	IRAP	GCATTGTTGTCACAGTCAAG	GCAAGTTACTCTTCATACCTGG	*Copia*	Lachesis_group2:990918-992537
Ma_LTR_139	repeat_TE1794557	RBIP	GATGACGATGATGCTCTG	GTGATTTGGCAGGCTTAC	*Copia*	Lachesis_group2:49005367-49006617
Ma_LTR_140	repeat_TE1794673	ISBP	ACTCTACTTATCCGCTGC	TCTCCTGTTTCTGACGAC	*Copia*	Lachesis_group2:49060974-49069094
Ma_LTR_141	repeat_TE1794746	IRAP	TCAATCACACACCCAGTG	TTCCAACATCTGAGAGCG	*Copia*	Lachesis_group2:49108815-49111119
Ma_LTR_142	repeat_TE1794746	RBIP	CAGTCTCCTTCTTCTTCAGA	CTCCATAGCAGAGCAAAG	*Copia*	Lachesis_group2:49108815-49111119
Ma_LTR_143	repeat_TE1794755	RBIP	GCTGGTCCTCTAACTAATAGC	TGTGTTGGTCTAATAGCCC	*Copia*	Lachesis_group2:49129093-49133810

Ma_LTR_144	repeat_TE1794755	IRAP	TCACCCAAATACAGGCAG	TGTGGTTAGCACCGAACT	*Copia*	Lachesis_group2:49129093-49133810
Ma_LTR_145	repeat_TE1794841	RBIP	GGTTACTTAGGCTCCAAGG	TCCTCAACAATCACCACC	*Copia*	Lachesis_group2:49200841-49202109
Ma_LTR_146	repeat_TE1794845	RBIP	ATCCCTTCTCTCCTTCCCT	TCACCTTGATACTTGCCG	*Copia*	Lachesis_group2:49204334-49205543
Ma_LTR_147	repeat_TE1794847	RBIP	GGTCTTCCTTTGCCTTTC	CCAAGTGGGAGAATGTTAGA	*Copia*	Lachesis_group2:49205645-49208811
Ma_LTR_148	repeat_TE1794858	IRAP	GGTGTGGACAGATAGTAAGG	GAGTTGGTAGGTTGAGTTTG	*Copia*	Lachesis_group2:49223605-49224355
Ma_LTR_149	repeat_TE1794869	RBIP	CTAAATGGAGGGAAGAGAGA	GTGACAACTTGAGTGCCA	*Copia*	Lachesis_group2:49227788-49229084
Ma_LTR_150	repeat_TE1795116	IRAP	GTCTTCCTCTTGCTTGGT	CGTCTATGTGGAGTAGTGG	*Copia*	Lachesis_group2:49380129-49381198
Ma_LTR_151	repeat_TE0000009	RBIP	CGTTTGAGTGTAGTATCGC	GTTGGTTCTTCTTGGGAG	*Gypsy*	Lachesis_group3:19917-24062
Ma_LTR_152	repeat_TE0000009	IRAP	CTTATCTCCCTCAACAAGC	CTACAGAAATGGCGACTTC	*Gypsy*	Lachesis_group3:19917-24062
Ma_LTR_153	repeat_TE0000009	IRAP	CAGCAACATAACGAGAACG	CCGAGAGAAATGAGAGAGAAGT	*Gypsy*	Lachesis_group3:19917-24062
Ma_LTR_154	repeat_TE0001566	RBIP	AGTAGGAACGAAACCAGG	CGTCTAAGTAAATGTGGGC	*Gypsy*	Lachesis_group3:651630-664788
Ma_LTR_155	repeat_TE0031021	RBIP	CTTGTTGCGTTAGTGTGC	AACTGGGATGGTCCGTAT	*Gypsy*	Lachesis_group3:12891314-12896300
Ma_LTR_156	repeat_TE0031115	RBIP	CATCAGAGTTCAAACGGAG	CGCTGGAGAAACCTATCA	*Gypsy*	Lachesis_group3:12932702-12947235
Ma_LTR_157	repeat_TE0031851	RBIP	CAAATACTGACTTGAGCGTC	GCGAGTGCTTTCTATGTC	*Gypsy*	Lachesis_group3:13269649-13272661
Ma_LTR_158	repeat_TE0048402	RBIP	GCGTAAGAACAACGAACG	TAAAGGAGGGTGCGGATA	*Gypsy*	Lachesis_group3:21142721-21145165
Ma_LTR_159	repeat_TE0048405	RBIP	CATTCTCTTCTCCTTCACTC	AGACCTCTCTTGCTCCTAC	*Gypsy*	Lachesis_group3:21144264-21145453
Ma_LTR_160	repeat_TE0048412	RBIP	GAAGAAGAAGGTGTGTGG	GGTGAGGTGTGAATAGCA	*Gypsy*	Lachesis_group3:21146984-21149604
Ma_LTR_161	repeat_TE0048414	IRAP	TTCATCTCTCCTCTCACTGT	CTTTCTTCCTTACATACGGG	*Gypsy*	Lachesis_group3:21147925-21154430

Ma_LTR_162	repeat_TE0048415	RBIP	TAGGGCATCCCGATAGAT	GCTCAAAGAGAAATAGCAGG	*Gypsy*	Lachesis_group3:21151979-21156511
Ma_LTR_163	repeat_TE0048415	RBIP	TTAGGACAATCCCGATGG	TGGAAGATGACTGCCTCA	*Gypsy*	Lachesis_group3:21151979-21156511
Ma_LTR_164	repeat_TE0048641	RBIP	TGGAAGATGACTGCCTCA	TTAGGACAATCCCGATGG	*Gypsy*	Lachesis_group3:21236649-21241845
Ma_LTR_165	repeat_TE0079303	RBIP	TTCCTTCCGACTCATCCT	CCTCAAGCAAACATCATACC	*Gypsy*	Lachesis_group3:38253144-38256025
Ma_LTR_166	repeat_TE0079303	RBIP	CAGGACCAATGAGTCACT	AAGAGTTCGTCTCCACAG	*Gypsy*	Lachesis_group3:38253144-38256025
Ma_LTR_167	repeat_TE0079307	RBIP	CTTTGACGAACTTGAACCC	CCACAGGGATTGTTTGGT	*Gypsy*	Lachesis_group3:38256122-38257578
Ma_LTR_168	repeat_TE0079334	RBIP	GGGTTATTGAACTTCTCCG	GATTGCCCTATGTCTCTTCT	*Gypsy*	Lachesis_group3:38262675-38266186
Ma_LTR_169	repeat_TE0079334	IRAP	GTGAATGTGTGTGTGTGC	CTTTGAGCAAGTGTGTAGGT	*Gypsy*	Lachesis_group3:38262675-38266186
Ma_LTR_170	repeat_TE0079399	RBIP	GTGACGAGAAGAAGAAAGG	CACAGATTTACCACTGGC	*Gypsy*	Lachesis_group3:38317704-38318286
Ma_LTR_171	repeat_TE0079524	RBIP	GAATGAGGGTTTCAGAGAGA	ATGCTTGGCAGTTACGAC	*Gypsy*	Lachesis_group3:38365465-38366316
Ma_LTR_172	repeat_TE0079534	RBIP	GGTCGGCATTCTTCCTTA	ACACTTCACAGGGACATCG	*Gypsy*	Lachesis_group3:38381575-38382079
Ma_LTR_173	repeat_TE0079534	RBIP	GGGAAGTCTCCAAGTTATC	TACGGGCACAATACTGAG	*Gypsy*	Lachesis_group3:38381575-38382079
Ma_LTR_174	repeat_TE0208480	RBIP	CAATGATGAGGAGATGAGAC	TCCAACAAGATAGCCAGG	*Gypsy*	Lachesis_group3:116395013-116397873
Ma_LTR_175	repeat_TE0208836	RBIP	CTAATGGTGTGATGGTGG	GAGAGAGGTTGGAGAGATG	*Gypsy*	Lachesis_group3:116541487-116543562
Ma_LTR_176	repeat_TE0208838	RBIP	GCCTTTATCTGTGGAACAC	GGTCTCATCAGTTTACCAAC	*Gypsy*	Lachesis_group3:116544208-116546809
Ma_LTR_177	repeat_TE0208838	IRAP	CGGATGCCTTTATCTGTG	CAAGCGGTCAAGTTCTCA	*Gypsy*	Lachesis_group3:116544208-116546809
Ma_LTR_178	repeat_TE0208839	IRAP	TCGCAACCAACTACACTG	CAAGACCACTCGCATCTA	*Gypsy*	Lachesis_group3:116546810-116554275
Ma_LTR_179	repeat_TE0209022	RBIP	CCAAAGGCTGTCTGTTAC	GGCTCTATGTTCTTGTCTCT	*Gypsy*	Lachesis_group4:12773-14933

Ma_LTR_180	repeat_TE0209023	RBIP	CCAAAGGCTGTCTGTTAC	GGCTCTATGTTCTTGTCTCT	*Gypsy*	Lachesis_group4:13022-15688
Ma_LTR_181	repeat_TE0209462	RBIP	CAAATCTTGGCACTAAGGC	GTTGTCGCATTGTTGGTC	*Gypsy*	Lachesis_group4:214124-216154
Ma_LTR_182	repeat_TE0209462	RBIP	CCTCTTCACATTCATCACC	TCACCATAACCAGGTAAGG	*Gypsy*	Lachesis_group4:214124-216154
Ma_LTR_183	repeat_TE0210498	RBIP	TTACTAATCCCACCACCC	GACGAAGGAGAAGAGAATG	*Gypsy*	Lachesis_group4:628949-631895
Ma_LTR_184	repeat_TE0210501	RBIP	TTACTAATCCCACCACCC	AGAAGAGACGAAGGAGAAG	*Gypsy*	Lachesis_group4:643630-648275
Ma_LTR_185	repeat_TE0210809	RBIP	AGGCTTGGCTTGATAGGA	CTGAAGACTTTGGAATCGC	*Gypsy*	Lachesis_group4:748045-749404
Ma_LTR_186	repeat_TE0210809	IRAP	TAGGCTTGGCTTGATAGG	GCTTCTCTGTTGCTGTGA	*Gypsy*	Lachesis_group4:748045-749404
Ma_LTR_187	repeat_TE0210829	IRAP	TGCTACAGACCAAGGGTA	GAGGAAGTTGAAACGGAC	*Gypsy*	Lachesis_group4:753364-754091
Ma_LTR_188	repeat_TE0210830	RBIP	AAGGAGGAGAAGAGGAAC	CATACCAGAATACACAGGG	*Gypsy*	Lachesis_group4:754092-755093
Ma_LTR_189	repeat_TE0293613	RBIP	CATTCACACTCTCGCTTC	GTTGTTTAGAACCCGACC	*Gypsy*	Lachesis_group4:40694816-40696583
Ma_LTR_190	repeat_TE0293614	RBIP	CACCACCTTTACCACACA	CACAGGGAACAAATCTGG	*Gypsy*	Lachesis_group4:40696584-40699040
Ma_LTR_191	repeat_TE0293615	RBIP	GGTGTGAATACCTTCCCT	CTAACCTACGAACATCTTGC	*Gypsy*	Lachesis_group4:40698517-40699202
Ma_LTR_192	repeat_TE0293622	RBIP	TTGTATCCAACGGGAACC	GCCAAGTGTTTGTCTTTCG	*Gypsy*	Lachesis_group4:40700315-40701035
Ma_LTR_193	repeat_TE0293630	RBIP	GAAGAGACAAAGAGCAAGG	CCAACCAGTGAAGTAGATTG	*Gypsy*	Lachesis_group4:40706342-40707335
Ma_LTR_194	repeat_TE0293630	IRAP	GAGGGAAAGAAGAGGCATA	CTAACCAATGAACAACTGGC	*Gypsy*	Lachesis_group4:40706342-40707335
Ma_LTR_195	repeat_TE0293631	IRAP	AACTGACCGCTTGCTAAGT	GCGAAGTGGCTTGTATCTT	*Gypsy*	Lachesis_group4:40706692-40708386
Ma_LTR_196	repeat_TE0293772	RBIP	GATTGTTCCGATTCAGGC	AGGACTTGCTGGATTTGG	*Gypsy*	Lachesis_group4:40777220-40777952
Ma_LTR_197	repeat_TE0293852	RBIP	GCCCTAAACACAAACCCT	TGCCTCACAAAGGATACG	*Gypsy*	Lachesis_group4:40804535-40805265

Ma_LTR_198	repeat_TE0294522	IRAP	GACTCGGGATTGTTGGTT	AGGTCTTGGGATTGTTGG	*Gypsy*	Lachesis_group4:41068815-41069501
Ma_LTR_199	repeat_TE0294526	RBIP	GTCTCCTCCAAGGCTAAA	GGTTCGCACTAAGTTTCC	*Gypsy*	Lachesis_group4:41081302-41083445
Ma_LTR_200	repeat_TE0294526	IRAP	GTCTCCTCCAAGGCTAAA	GTCTTTGAGCAAGTGTGC	*Gypsy*	Lachesis_group4:41081302-41083445
Ma_LTR_201	repeat_TE0294527	RBIP	AACAAGGTAAGGGTGGGA	TTCACTTTGGGAGGCTGT	*Gypsy*	Lachesis_group4:41083421-41087793
Ma_LTR_202	repeat_TE0294871	IRAP	AACATTGTCGGTCTCCTG	GTAAATCACTCGCTTCTACG	*Gypsy*	Lachesis_group4:41180799-41186325
Ma_LTR_203	repeat_TE0295297	RBIP	AGAGGTTGAAAGAGGTGTC	GAGGTGTGAATAGCAGAAC	*Gypsy*	Lachesis_group4:41370987-41373481
Ma_LTR_204	repeat_TE0295305	RBIP	TGTTCTGGCTATGAGACAGC	CATTGAGAGTGGGATTCGT	*Gypsy*	Lachesis_group4:41386362-41395100
Ma_LTR_205	repeat_TE0295307	RBIP	AAAGTAGGCTCAAGAGGG	CCTGTTCCGATGTAGTTAC	*Gypsy*	Lachesis_group4:41394690-41400203
Ma_LTR_206	repeat_TE0353401	RBIP	GTCTTCTACTCTCCAACTTCTC	GAGCACCACAAATCACAC	*Gypsy*	Lachesis_group4:83582356-83583290
Ma_LTR_207	repeat_TE0353725	RBIP	TCTGACACAATACTGGAAGG	TGTTGATAGGTTGGGTGG	*Gypsy*	Lachesis_group4:83762377-83763840
Ma_LTR_208	repeat_TE0353726	RBIP	CAACTCCTTCTCCTTCAAGA	AGGGCTCTGATTAGGTGTC	*Gypsy*	Lachesis_group4:83763841-83767896
Ma_LTR_209	repeat_TE0396587	RBIP	GTCTCACACACAAGATTCC	GGTGGTTAGGGAGGTTAT	*Gypsy*	Lachesis_group5:170188-171008
Ma_LTR_210	repeat_TE0396588	RBIP	GTCTCACACACAAGATTCC	GGTGGTTAGGGAGGTTAT	*Gypsy*	Lachesis_group5:170601-171654
Ma_LTR_211	repeat_TE0397116	RBIP	GTTCTCTGAAAGGAGCCA	TATCGTCCACAGGGAGTT	*Gypsy*	Lachesis_group5:337823-339134
Ma_LTR_212	repeat_TE0396215	RBIP	AGCCAAAGGTAAAGTCCC	CACATAATGGTTGACCCG	*Gypsy*	Lachesis_group5:3863-5697
Ma_LTR_213	repeat_TE0398079	RBIP	AGAGAATCACCTTGTCCTC	AACTTCCTCCTCACTCCA	*Gypsy*	Lachesis_group5:752267-754252
Ma_LTR_214	repeat_TE0398081	RBIP	CTGGAGTTTGGTGAAAGTC	CCTTAGTTGGTAGTTTAGCC	*Gypsy*	Lachesis_group5:755969-756972
Ma_LTR_215	repeat_TE0398081	IRAP	CGTCGTGACCATCAAATC	AGGAACATACGCAAACCC	*Gypsy*	Lachesis_group5:755969-756972

Ma_LTR_216	repeat_TE0398129	RBIP	TTCTCACCGAAGTCACCT	CCGTTTGTGTTTACCCTC	*Gypsy*	Lachesis_group5:765548-769940
Ma_LTR_217	repeat_TE0398129	RBIP	CATCGCACTTTGAGGATAC	TAGCCAACACTACGCAAC	*Gypsy*	Lachesis_group5:765548-769940
Ma_LTR_218	repeat_TE0398131	RBIP	GAACCCGACTTTGATTGC	AGGTGCCCAGAGTTATGAG	*Gypsy*	Lachesis_group5:767283-770416
Ma_LTR_219	repeat_TE0398142	RBIP	CCCACGATTTCACTGACA	TACTGGTTGAGACCCGCTA	*Gypsy*	Lachesis_group5:793474-801615
Ma_LTR_220	repeat_TE0398142	IRAP	GAAGGAATCAAGGCAGGA	CCCTCAAACCATAGCAATG	*Gypsy*	Lachesis_group5:793474-801615
Ma_LTR_221	repeat_TE0520551	RBIP	TACAACGGATGTGAGGGA	TTCAGTAAAGTCGCCACC	*Gypsy*	Lachesis_group5:54917581-54919113
Ma_LTR_222	repeat_TE0520552	IRAP	AAACCAGAGTCTCCGTGA	CCAGGGATAACCTTCAAAC	*Gypsy*	Lachesis_group5:54919114-54922681
Ma_LTR_223	repeat_TE0520555	RBIP	TGTCCCGTCCTCAGATACT	CATCCCTCACATTCGTTG	*Gypsy*	Lachesis_group5:54925592-54928054
Ma_LTR_224	repeat_TE0520572	IRAP	GATAGGGTTGCTGTTGGT	AGGTAGAGGAAAGTGGCA	*Gypsy*	Lachesis_group5:54932938-54935564
Ma_LTR_225	repeat_TE0520601	RBIP	GTCAACTTTCAGAGAATGCC	GGTTTGGTCTTGTTCCCA	*Gypsy*	Lachesis_group5:54953706-54956623
Ma_LTR_226	repeat_TE0520633	RBIP	GCTTCAAGTGTGGTGGAT	AACGCAACCCTTCTCTCT	*Gypsy*	Lachesis_group5:54978052-54978722
Ma_LTR_227	repeat_TE0572157	RBIP	AAGGAGATAGAGGTGGTGG	GGTGGGAATAGCAAGGTT	*Gypsy*	Lachesis_group5:91970128-91971439
Ma_LTR_228	repeat_TE0572167	IRAP	AGAGAAAGGAGAGGGAGA	CATACGCACACACACTTC	*Gypsy*	Lachesis_group5:91975239-91977241
Ma_LTR_229	repeat_TE0572169	RBIP	ATCGGAATGGACTCTACC	GTGTATGCGTATGTGTGAG	*Gypsy*	Lachesis_group5:91987718-91989737
Ma_LTR_230	repeat_TE0572170	IRAP	AGAGAAAGGAGAGGGAGA	CATACGCACACACACTTC	*Gypsy*	Lachesis_group5:91989453-91991531
Ma_LTR_231	repeat_TE0572177	RBIP	CTTGGAGGAGACAAAGTTC	CACAACATCTTGGCTCTG	*Gypsy*	Lachesis_group5:92005013-92006908
Ma_LTR_232	repeat_TE0572178	RBIP	CTTGGAGGAGACAAAGTTC	CACAACATCTTGGCTCTG	*Gypsy*	Lachesis_group5:92005017-92007021
Ma_LTR_233	repeat_TE0572216	RBIP	ATGTTAGTGGTCTCCAGC	CGAGGATACAGAGGGATAG	*Gypsy*	Lachesis_group5:92016830-92020003

Ma_LTR_234	repeat_TE0572229	ISBP	AAGAGGTTCCCGTAGAGT	GACACTTTGATTAGACCACC	*Gypsy*	Lachesis_group5:92025156-92029165
Ma_LTR_235	repeat_TE0572230	IRAP	CACCCTTGTAAATCCCAG	GATGTCTTGAGTTGGAGGT	*Gypsy*	Lachesis_group5:92029153-92032415
Ma_LTR_236	repeat_TE0572232	IRAP	CATCAGTCCACTTCCAGTC	TGGGTGAGCAGACACTTA	*Gypsy*	Lachesis_group5:92031268-92034351
Ma_LTR_237	repeat_TE0572233	IRAP	CCCAGTGAAACTACATCATC	TAATGCCCTTGGTCTCAG	*Gypsy*	Lachesis_group5:92032692-92034311
Ma_LTR_238	repeat_TE0572235	RBIP	TCTACCCAGAACTACGAGG	GTGCTTCAACAGTGACCT	*Gypsy*	Lachesis_group5:92035668-92036812
Ma_LTR_239	repeat_TE0572243	RBIP	CCTGTGGGCTTGATTTAG	CACCTCATCTCCAAACTACA	*Gypsy*	Lachesis_group5:92049696-92052924
Ma_LTR_240	repeat_TE0572244	RBIP	GGTAAGATGTCGCCCTAT	TTCAGCAGCAGTAAGTGG	*Gypsy*	Lachesis_group5:92050152-92053260
Ma_LTR_241	repeat_TE0653017	RBIP	GAAGAGAGAGAACCGAGAA	TTGGGTAAAGAGAGTCCG	*Gypsy*	Lachesis_group6:120046-122798
Ma_LTR_242	repeat_TE0653018	IRAP	CAAAGCATCTGACATCCAC	CAAACGCATCATCTCTCG	*Gypsy*	Lachesis_group6:122410-127080
Ma_LTR_243	repeat_TE0653019	RBIP	AAGAGTCAAACCTCACCC	CTGTTGCTGATACCGTTG	*Gypsy*	Lachesis_group6:122799-124535
Ma_LTR_244	repeat_TE0653019	IRAP	ATCACTTCCAAGGGTCTG	TAAGGAGGAGGGATTGTG	*Gypsy*	Lachesis_group6:122799-124535
Ma_LTR_245	repeat_TE0653021	IRAP	TTTACTTCTTGGGCGTCC	CTTCACAATGTTGCCTCG	*Gypsy*	Lachesis_group6:127092-131425
Ma_LTR_246	repeat_TE0653023	RBIP	AGTTTCACACACCAGTGG	CGTCGTAGGAATCTAACAC	*Gypsy*	Lachesis_group6:131433-132585
Ma_LTR_247	repeat_TE0653027	RBIP	CACACACACCAAACTGAAAC	CGGTTCTCTCTCTTCTCTCA	*Gypsy*	Lachesis_group6:133264-133984
Ma_LTR_248	repeat_TE0653140	IRAP	TCCTCACTATCCTCATCGG	TGGAAGAAAGAAGGGCTC	*Gypsy*	Lachesis_group6:207926-217401
Ma_LTR_249	repeat_TE0653145	IRAP	AGAGGAAGGTATTCGCAC	GTAGGAGCAAGAGAGGTCT	*Gypsy*	Lachesis_group6:217624-218707
Ma_LTR_250	repeat_TE0653566	IRAP	CCAACCAATAACACCCTC	TAGGACAACAACCAGTGC	*Gypsy*	Lachesis_group6:370829-373866
Ma_LTR_251	repeat_TE0653570	IRAP	CCTAAATGTTGGCTCCAGT	GGTGACGATTCAAAGTGTC	*Gypsy*	Lachesis_group6:374502-375356

Ma_LTR_252	repeat_TE0654071	IRAP	CTTCCCAACTTCCAATCG	TGTATTAGGATGGCACCG	*Gypsy*	Lachesis_group6:529055-530061
Ma_LTR_253	repeat_TE0654088	RBIP	TCACATCAGCAGTTACTCC	GGTTGTAGCCTGTCAAGTC	*Gypsy*	Lachesis_group6:537620-539623
Ma_LTR_254	repeat_TE0654130	RBIP	AGCGATTCTAAGACGACG	GCTTGATGCTCTGATGGT	*Gypsy*	Lachesis_group6:575905-581234
Ma_LTR_255	repeat_TE0654380	RBIP	ACTTGCTATGACTGCTCCG	TTAGGACAATCCCGATGG	*Gypsy*	Lachesis_group6:668547-674072
Ma_LTR_256	repeat_TE0687518	IRAP	GAAGAGGAGAAGAATGGGT	GAGGGATTGTGATGCTAAAC	*Gypsy*	Lachesis_group6:17354756-17359902
Ma_LTR_257	repeat_TE0687518	IRAP	GAGTCTTTGTTCCCTTCC	CCCTGGGTTGAGATAGTT	*Gypsy*	Lachesis_group6:17354756-17359902
Ma_LTR_258	repeat_TE0687519	IRAP	GTCTTTGTTCCCTTCCTG	CCTGGGTTGAGATAGTTG	*Gypsy*	Lachesis_group6:17359140-17360013
Ma_LTR_259	repeat_TE0687549	RBIP	AAGTCCCTGTGGATTCTC	GTCCTATCATACACGCCA	*Gypsy*	Lachesis_group6:17373512-17374604
Ma_LTR_260	repeat_TE0687586	RBIP	GGGAGTGATGAGAGAGAA	GTCAAAGAGTTTACTGGTCC	*Gypsy*	Lachesis_group6:17412646-17415088
Ma_LTR_261	repeat_TE0687600	IRAP	AGGAATCTCACCACTTGG	GTTTCACACACTTTCGGTC	*Gypsy*	Lachesis_group6:17421098-17428157
Ma_LTR_262	repeat_TE0687601	RBIP	GGTTAGAACTACGGCTTTG	TCCCTACAAGGATTGACC	*Gypsy*	Lachesis_group6:17428158-17428944
Ma_LTR_263	repeat_TE0687965	RBIP	TCTAAGGTAAGGGTGGGA	CTCAACCAACCAACATCTG	*Gypsy*	Lachesis_group6:17599056-17601064
Ma_LTR_264	repeat_TE0688011	IRAP	AGGCTTGGCTTGATAGGA	CTGAAGACTTTGGAATCGC	*Gypsy*	Lachesis_group6:17614753-17616818
Ma_LTR_265	repeat_TE0688024	RBIP	GACACAACAACACCGACT	GGTCCTAACCTACCAAATG	*Gypsy*	Lachesis_group6:17624889-17626891
Ma_LTR_266	repeat_TE0688144	IRAP	TCGCACTTTGAGGATACG	ATTCGGGAGTCGGTTATG	*Gypsy*	Lachesis_group6:17734501-17736062
Ma_LTR_267	repeat_TE0688145	IRAP	ACACCGAAGGACTTAGACC	CACGGACCAGAAACTATG	*Gypsy*	Lachesis_group6:17736063-17743643
Ma_LTR_268	repeat_TE0688148	IRAP	TCGCACTTTGAGGATACG	ATTCGGGAGTCGGTTATG	*Gypsy*	Lachesis_group6:17743663-17744189
Ma_LTR_269	repeat_TE0688282	RBIP	GAACTATTGTTGGACCACC	GGACTCAAGGACTCATCTAA	*Gypsy*	Lachesis_group6:17796688-17797272

Ma_LTR_270	repeat_TE0701835	RBIP	TACTCAGGGCAGGAAGTTC	GAAGAAGAGGAAGGCGAT	*Gypsy*	Lachesis_group6:25680571-25682574
Ma_LTR_271	repeat_TE0701861	RBIP	TCCGTAGGAACCTCTTTG	TCCGCTTTGACTTGTGTC	*Gypsy*	Lachesis_group6:25696778-25698842
Ma_LTR_272	repeat_TE0701862	RBIP	CTGATTGGACTCTTGGACAT	ATTTCGGCAGGGAGGTAT	*Gypsy*	Lachesis_group6:25698843-25699358
Ma_LTR_273	repeat_TE0737657	IRAP	CTTCCCTTAGCGTAACCA	GGACTTATCTCCCTCAACA	*Gypsy*	Lachesis_group0:107325-109982
Ma_LTR_274	repeat_TE0737658	IRAP	ACCTTCCCTTAGCGTAAC	ATGTGTCACTCAAGTCCG	*Gypsy*	Lachesis_group0:107983-114174
Ma_LTR_275	repeat_TE0737663	RBIP	CCCTGTCCCTTGGATAAA	GCGTTTCTTTCTCTCCTTC	*Gypsy*	Lachesis_group0:117471-118273
Ma_LTR_276	repeat_TE0737964	IRAP	GGAAGAAGGAGGACAAAG	CAACACACATACGCCACT	*Gypsy*	Lachesis_group0:217965-220031
Ma_LTR_277	repeat_TE0737967	RBIP	TCAGATGGAGTTGTGAGG	GAGGCTAAACCCTACGAT	*Gypsy*	Lachesis_group0:220033-221772
Ma_LTR_278	repeat_TE0738157	ISBP	AAGAGGTTCCTACGGAGTA	CCAGAGTTATGGGTTCAAC	*Gypsy*	Lachesis_group0:282221-284374
Ma_LTR_279	repeat_TE0738158	RBIP	GAACTGTATGTGTCCAAGG	CCAGGAAGAGAACAAGAC	*Gypsy*	Lachesis_group0:284356-285902
Ma_LTR_280	repeat_TE0738159	RBIP	GAACTGTATGTGTCCAAGG	CCAGGAAGAGAACAAGAC	*Gypsy*	Lachesis_group0:284578-285933
Ma_LTR_281	repeat_TE0738167	RBIP	AGAGGAAGAAGACAACCG	GTCACAAAGGATGAGGGT	*Gypsy*	Lachesis_group0:299198-301333
Ma_LTR_282	repeat_TE0765130	IRAP	TCTTCCAGGTTTCTCTGC	CCCGACACAAGGTAGTAAT	*Gypsy*	Lachesis_group0:11636132-11641275
Ma_LTR_283	repeat_TE0765739	RBIP	CCCGAATCTAAGGTCAAAGT	CACGCAAGAAACACATCAC	*Gypsy*	Lachesis_group0:11895199-11897391
Ma_LTR_284	repeat_TE0765740	IRAP	ATTTGGACCAGGCACACT	AAGCACTCCGTCATCGTA	*Gypsy*	Lachesis_group0:11896851-11899384
Ma_LTR_285	repeat_TE0765742	RBIP	CAACTAACGAATCGCTGAGT	GGAAGAAGAGAATGTCGGA	*Gypsy*	Lachesis_group0:11902822-11909811
Ma_LTR_286	repeat_TE0765907	IRAP	CGGATGATACGAAAGTGAG	GCTTCTGTTGTTAGCCCAT	*Gypsy*	Lachesis_group0:11952710-11954058
Ma_LTR_287	repeat_TE0765911	RBIP	AAGTGGACCGACATCACA	ATGACGACATCTGCCAAG	*Gypsy*	Lachesis_group0:11954727-11958976

Ma_LTR_288	repeat_TE0765916	IRAP	ACCCTCTTGGAGTGATACT	TTCTACCACGAACTACGAG	*Gypsy*	Lachesis_group0:11975466-11977861
Ma_LTR_289	repeat_TE0765979	RBIP	GGTGAGAGAACCGAGTTT	TGGAGTTTCGTAGAGACG	*Gypsy*	Lachesis_group0:11993134-11994012
Ma_LTR_290	repeat_TE0793015	RBIP	ACTAAGGTTCCAGGCTGT	GACTCATCCAACAATCCC	*Gypsy*	Lachesis_group0:24664021-24665133
Ma_LTR_291	repeat_TE0793019	RBIP	CTGGATTGAGAGAATACGG	ACAAACAGTTCCCTACCC	*Gypsy*	Lachesis_group0:24666775-24670495
Ma_LTR_292	repeat_TE0793030	RBIP	GCAACTTTACATTCCAGCAC	CAACCTTATTGTCCCAAGAC	*Gypsy*	Lachesis_group0:24684324-24685510
Ma_LTR_293	repeat_TE0793128	RBIP	CGGCAAGGTAGAGAGAAGT	AATGGGCTTTGGAGTAGG	*Gypsy*	Lachesis_group0:24725739-24727786
Ma_LTR_294	repeat_TE0834252	IRAP	GTTTAGAACCTGACCGCT	GCTCCATCAAGTCCATCT	*Gypsy*	Lachesis_group0:49878309-49881825
Ma_LTR_295	repeat_TE0834258	RBIP	TGTTGAGTAGCAAGGCAG	TAGGCAATAGAGAGCGTC	*Gypsy*	Lachesis_group0:49884282-49886587
Ma_LTR_296	repeat_TE0834263	IRAP	GGTGTAGGACTTATCTCACTC	ACCTTCCCTTAGCGTAAC	*Gypsy*	Lachesis_group0:49889163-49892955
Ma_LTR_297	repeat_TE0834415	IRAP	CAGGACTATCTCACCACTCA	GACAGCAATGACAACTTCAC	*Gypsy*	Lachesis_group0:49973239-49974459
Ma_LTR_298	repeat_TE0834416	IRAP	CAGGACTATCTCACCACTCA	GACAGCAATGACAACTTCAC	*Gypsy*	Lachesis_group0:49973241-49974446
Ma_LTR_299	repeat_TE0834418	RBIP	GTGAGCACCAAGAGTGTAT	GATAGAAGTGGGATGAAGG	*Gypsy*	Lachesis_group0:49974652-49975226
Ma_LTR_300	repeat_TE0834478	IRAP	CTCTCACACATACACAAAGG	ATCTGGAGTTCTGGAAGTC	*Gypsy*	Lachesis_group0:49991246-49992031
Ma_LTR_301	repeat_TE1457295	RBIP	CAAGCACGGTAAGTTAGC	CGAGTTCAAGAGCACCTT	*Gypsy*	Lachesis_group1:125980-132007
Ma_LTR_302	repeat_TE1457731	RBIP	AAAGAGGACAAAGACCCG	GCGTATGTGTGTTGACTA	*Gypsy*	Lachesis_group1:280665-289952
Ma_LTR_303	repeat_TE1457734	IRAP	ATGTCACTCCCATACTCAC	AACTGCTCATCTGTAGCC	*Gypsy*	Lachesis_group1:291734-299236
Ma_LTR_304	repeat_TE1482806	IRAP	GGGTCTTCAACCTATCAGG	CCTCCTTACAAATCCCAG	*Gypsy*	Lachesis_group1:14805660-14818268
Ma_LTR_305	repeat_TE1482851	RBIP	TAATGACACAGGGAGCCA	GAGTCGTTGATGTTGCCT	*Gypsy*	Lachesis_group1:14851947-14854121

Ma_LTR_306	repeat_TE1482902	RBIP	TACTCGGAAGAGCGTTCT	GTATCTCCTCAATGGATGC	*Gypsy*	Lachesis_group1:14894444-14895870
Ma_LTR_307	repeat_TE1511354	RBIP	GGTTGTTGTGTAGAAGCG	AGTATTGAGCCACCAAGAC	*Gypsy*	Lachesis_group1:32982365-32983758
Ma_LTR_308	repeat_TE1511454	IRAP	GACAAGAGAGACAAGTTCAGAG	AAAGACACACACGCACAC	*Gypsy*	Lachesis_group1:33113943-33115607
Ma_LTR_309	repeat_TE1511762	IRAP	CACACACACACACACTCAT	TGTTTACTCCACTGGGTC	*Gypsy*	Lachesis_group1:33227320-33228072
Ma_LTR_310	repeat_TE1511770	IRAP	ATAGAACCTGACCGCTTG	CATACGCATACACAACGC	*Gypsy*	Lachesis_group1:33231252-33232505
Ma_LTR_311	repeat_TE1523154	IRAP	ATCACTTCCAAGGGTCTG	GAGGAGGGATTGTAATGCT	*Gypsy*	Lachesis_group1:41821007-41829360
Ma_LTR_312	repeat_TE1523188	RBIP	TATCTTTGACCCGTGGAG	AAATAGGGACAAGGGACG	*Gypsy*	Lachesis_group1:41861093-41862403
Ma_LTR_313	repeat_TE1575724	IRAP	TGATTGCCTCTGTTACCC	TTGTCTACTACCGATGCG	*Gypsy*	Lachesis_group1:75391992-75393231
Ma_LTR_314	repeat_TE1575732	RBIP	CATCAGCAAGTAATGCGG	GCCCTTATGTGGAAATAGG	*Gypsy*	Lachesis_group1:75411765-75415494
Ma_LTR_315	repeat_TE1575753	IRAP	AGCCACCTTATCCACTACG	CTCAAATCCGACCCTACA	*Gypsy*	Lachesis_group1:75431420-75433647
Ma_LTR_316	repeat_TE1575759	RBIP	GCGGTGTAGTTTCACTCAT	CATCCTCTAAGATTGGGAC	*Gypsy*	Lachesis_group1:75438749-75442662
Ma_LTR_317	repeat_TE1575788	RBIP	TTGTTTGAGGACGAGGTC	CCGTAGGAAGCCATACAT	*Gypsy*	Lachesis_group1:75463065-75465331
Ma_LTR_318	repeat_TE1575813	RBIP	CTGTATGTATCCCAACGAC	TATGGAGCCCTTAGGAAG	*Gypsy*	Lachesis_group1:75480485-75481402
Ma_LTR_319	repeat_TE1575833	RBIP	TTGTCTACTACCGATGCG	TGATTGCCTCTGTTACCC	*Gypsy*	Lachesis_group1:75491208-75492446
Ma_LTR_320	repeat_TE1605205	ISBP	TCTCGTCAAGAGGTGTCAC	TGGTGTAAGATGGCGAAG	*Gypsy*	Lachesis_group1:91847762-91853114
Ma_LTR_321	repeat_TE1605206	ISBP	GAGGATTCCCAACATCTTAG	GGTCTCGGTGAAAGTGTAG	*Gypsy*	Lachesis_group1:91847885-91852213
Ma_LTR_322	repeat_TE1605315	ISBP	CGTTTCAGTAATCCACCC	TTTCTGGCGAGTTGTCAC	*Gypsy*	Lachesis_group1:91882513-91891826
Ma_LTR_323	repeat_TE1628673	RBIP	TTAGGACAATCCCGATGG	TACTGGAATGGACCGTGA	*Gypsy*	Lachesis_group1:102933349-102938515

Ma_LTR_324	repeat_TE1628927	IRAP	GGTATCAGAGCCTGGTTAG	AAACAGTCCTCAGTTCCTC	*Gypsy*	Lachesis_group1:103054781-103059320
Ma_LTR_325	repeat_TE1628928	IRAP	GAGTGAATCCTGTTACCG	CTGTCAAGTCTCATTTCCTC	*Gypsy*	Lachesis_group1:103059321-103063429
Ma_LTR_326	repeat_TE1740644	RBIP	CGGGAAATCTCCACAACT	GAAGGGAAATGGTAGCATC	*Gypsy*	Lachesis_group2:17314306-17317740
Ma_LTR_327	repeat_TE1740716	RBIP	TCTACTTCTCTCTCAGGCG	CGTGTGACGGTTGAATAC	*Gypsy*	Lachesis_group2:17338120-17344094
Ma_LTR_328	repeat_TE1740793	IRAP	ACACACGCACACACACTCT	CAGCCCAGTTCCTTGTTTA	*Gypsy*	Lachesis_group2:17385375-17388963
Ma_LTR_329	repeat_TE1740795	IRAP	CCTGTCTAAAGTCGCAAG	CAGAAGATGGTGGTCACT	*Gypsy*	Lachesis_group2:17388731-17395516
Ma_LTR_330	repeat_TE1740796	RBIP	GTTTCGGTTCTGATGGTG	ACAAATCCACACACAGCC	*Gypsy*	Lachesis_group2:17395517-17396693
Ma_LTR_331	repeat_TE1741039	RBIP	CTAATGCCTCCCAGTTTAC	GAGTTGTCAGTAGTATGGACC	*Gypsy*	Lachesis_group2:17471682-17475434
Ma_LTR_332	repeat_TE1741050	ISBP	CGATAAGTCGCAAACCTC	TAGGATGAAACAGTGCCC	*Gypsy*	Lachesis_group2:17490624-17495073
Ma_LTR_333	repeat_TE1741052	RBIP	CGACATTCGTCCTCAACA	ACTCAACGGGAGCCATTA	*Gypsy*	Lachesis_group2:17496772-17498108
Ma_LTR_334	repeat_TE1741356	IRAP	GTAAACTGGAGGTGTGGATA	GAGGAAGAAGAGAGAGGAAT	*Gypsy*	Lachesis_group2:17596876-17598364
Ma_LTR_335	repeat_TE1792011	RBIP	TGCGTTACCTGAGACCAT	AAGTGTTAGCCTGGACTGG	*Gypsy*	Lachesis_group2:47357582-47361917
Ma_LTR_336	repeat_TE1792027	IRAP	GAGGAAGTAGACGCTTATTG	GTTGGTGGTGTCATTCAC	*Gypsy*	Lachesis_group2:47375213-47378283
Ma_LTR_337	repeat_TE1792032	RBIP	AGCAAAGTAGGGACGACT	TGATAGTGACAACTCCGC	*Gypsy*	Lachesis_group2:47379502-47381080
Ma_LTR_338	repeat_TE1819088	RBIP	CATAACTGACTCCCGAAC	CACCGACTATCCCTAATG	*Gypsy*	Lachesis_group2:65998091-66004471
Ma_LTR_339	repeat_TE1819089	IRAP	GCTACAATACTCGGCTAAAC	GTCATTACTCGCAACACC	*Gypsy*	Lachesis_group2:66001587-66004709
Ma_LTR_340	repeat_TE1819098	IRAP	ACCTTCCCTTAGCGTAAC	CCGTTCCTATCTTATGTGTC	*Gypsy*	Lachesis_group2:66015781-66022059
Ma_LTR_341	repeat_TE1819129	RBIP	GACTTACCCAACAATCCC	CTCTCAAATCTCACTGGC	*Gypsy*	Lachesis_group2:66042537-66043527

Ma_LTR_342	repeat_TE1819279	IRAP	TGAGTCTTGAGTCCCTTG	GATTACTGGTATGCCCTTC	*Gypsy*	Lachesis_group2:66124859-66127312
Ma_LTR_343	repeat_TE1853215	IRAP	TGTCCTTGTTGGGTATCC	GTAAAGTTGATGCGGAGTC	*Gypsy*	Lachesis_group2:88956367-88960693
Ma_LTR_344	repeat_TE1860476	RBIP	TATGAGGTCTCTGCCCTT	GCTGCTTGCTGATTTAGG	*Gypsy*	Lachesis_group2:93050820-93052081
Ma_LTR_345	repeat_TE1860478	IRAP	TCCTCCTGAGAATACCCT	AGAGTGCTACTTGTTGGG	*Gypsy*	Lachesis_group2:93051721-93055869
Ma_LTR_346	repeat_TE1860489	RBIP	GCAAGTCCCAGAGAAGAT	CCTAATACATCAGACAAGGC	*Gypsy*	Lachesis_group2:93076951-93081719
Ma_LTR_347	repeat_TE1860511	IRAP	GACTCATCCAACAATCCC	GAAGGAATCTGGCTAAAGC	*Gypsy*	Lachesis_group2:93122525-93123818
Ma_LTR_348	repeat_TE1860639	RBIP	CAAACTTGGCATTACAGGC	TCGTCTTAGGAGTCGCTCT	*Gypsy*	Lachesis_group2:93176983-93180527
Ma_LTR_349	repeat_TE1860968	RBIP	GCGGGACTATCATTCTCT	CAACAGCCTATTCATCTGAC	*Gypsy*	Lachesis_group2:93425265-93427252
Ma_LTR_350	repeat_TE1860986	RBIP	TCACAGAGTTTGAGTCCC	GAAGAAGAAGGTGGGTTC	*Gypsy*	Lachesis_group2:93436681-93438161
