# Supplementary figures and images for: Genome-Wide Identification and Development of LTR Retrotransposon-Based Molecular Markers for the Melilotus Genus
Source: Plants (Basel). 2021 Apr 28;10(5):890. doi: 10.3390/plants10050890 (PMC8146837; doi:10.3390/plants10050890)

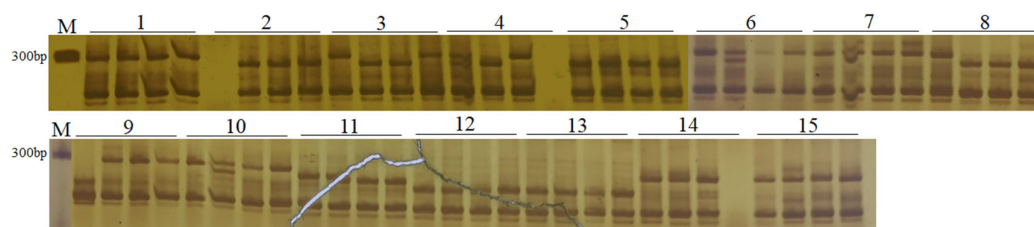

Primer 25\_RBIP

Supplement: Supplementary file 1 [file plants-10-00890-s001.zip › Supplementary/Figure S1.pdf]

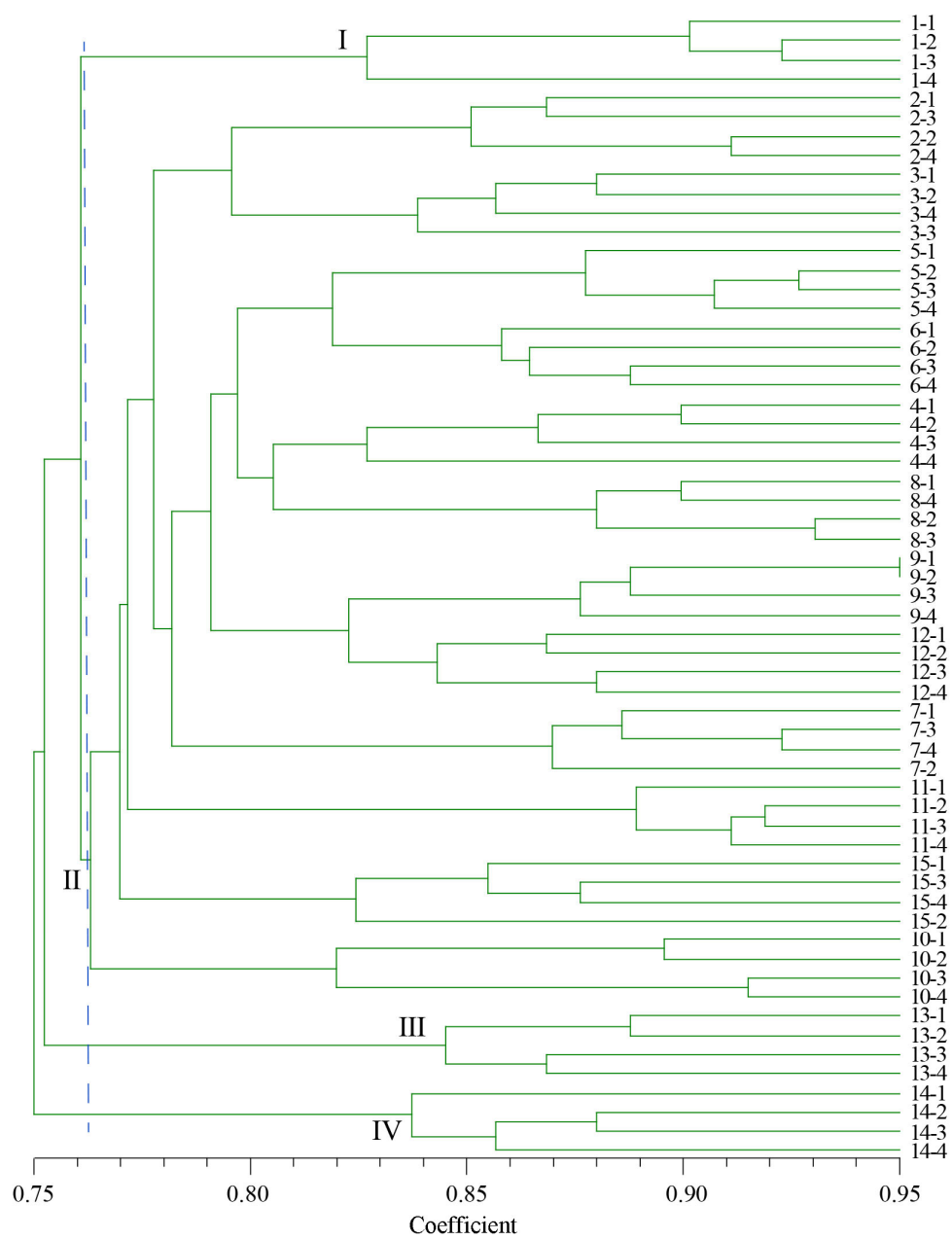

Supplement: Supplementary file 1 [file plants-10-00890-s001.zip › Supplementary/Figure S2.pdf]
